# A prophylactic α-Gal-based glycovaccine effectively protects against murine acute Chagas disease

**DOI:** 10.1038/s41541-019-0107-7

**Published:** 2019-03-22

**Authors:** Susana Portillo, Brenda G. Zepeda, Eva Iniguez, Janet J. Olivas, Nasim H. Karimi, Otacilio C. Moreira, Alexandre F. Marques, Katja Michael, Rosa A. Maldonado, Igor C. Almeida

**Affiliations:** 1 Department of Biological Sciences, Border Biomedical Research Center, The University of Texas at El Paso, El Paso, TX USA; 2 Laboratório de Biologia Molecular e Doenças Endêmicas, Fundação Instituto Oswaldo Cruz (Fiocruz), Rio de Janeiro, Rio de Janeiro Brazil; 3 Departamento de Parasitologia, Instituto de Ciências Biológicas, Universidade Federal de Minas Gerais, Belo Horizonte, MG 31270-901 Brazil; 4 Department of Chemistry and Biochemistry, Border Biomedical Research Center, The University of Texas at El Paso, El Paso, TX USA

## Abstract

Chagas disease (ChD), caused by the hemoflagellate parasite *Trypanosoma cruzi*, affects six to seven million people in Latin America. Lately, it has become an emerging public health concern in nonendemic regions such as North America and Europe. There is no prophylactic or therapeutic vaccine as yet, and current chemotherapy is rather toxic and has limited efficacy in the chronic phase of the disease. The parasite surface is heavily coated by glycoproteins such as glycosylphosphatidylinositol (GPI)-anchored mucins (tGPI-mucins), which display highly immunogenic terminal nonreducing α-galactopyranosyl (α-Gal)-containing glycotopes that are entirely absent in humans. The immunodominant tGPI-mucin α-Gal glycotope, the trisaccharide Galα1,3Galβ1,4GlcNAc (Galα3LN), elicits high levels of protective *T*. *cruzi*-specific anti-α-Gal antibodies in ChD patients in both the acute and chronic phases. Although glycoconjugates are the major parasite glycocalyx antigens, they remain completely unexplored as potential ChD vaccine candidates. Here we investigate the efficacy of the *T*. *cruzi* immunodominant glycotope Galα3LN, covalently linked to a carrier protein (human serum albumin (HSA)), as a prophylactic vaccine candidate in the acute model of ChD, using the α1,3-galactosyltransferase-knockout (α1,3GalT-KO) mouse, which mimics the human immunoresponse to α-Gal glycotopes. Animals vaccinated with Galα3LN-HSA were fully protected against lethal *T*. *cruzi* challenge by inducing a strong anti-α-Gal antibody-mediated humoral response. Furthermore, Galα3LN-HSA-vaccinated α1,3GalT-KO mice exhibited significant reduction (91.7–99.9%) in parasite load in all tissues analyzed, cardiac inflammation, myocyte necrosis, and T cell infiltration. This is a proof-of-concept study to demonstrate the efficacy of a prophylactic α-Gal-based glycovaccine for experimental acute Chagas disease.

## Chagas disease: Unexplored glycans make for an effective anti-parasitic therapy

A vaccine candidate derived from an immunodominant parasitic glycan could offer a much-needed preventive therapy for Chagas disease. The disease, caused by the parasite *Trypanosoma cruzi*, is endemic to Latin America and an emergent threat to North America and Europe. Current therapies are few, poorly efficacious, and toxic. Igor Almeida, from the University of Texas at El Paso, United States, and his team created a candidate which presents a host with *T*. *cruzi* surface-derived α-galactose-containing (α-Gal) glycan covalently linked to a carrier protein. Parasite-derived α-Gal-containing proteins are known to be highly immune-stimulating to humans but were previously unexplored as prophylactics. In a mouse model designed to mimic the human response to Chagas disease, vaccinated animals had a strong antibody response and were fully protected against lethal exposure to *T*. *cruzi*. The results offer a promising candidate for future research and validate the method used in this proof-of-concept study.

## Introduction

Chagas disease (ChD), caused by the protozoan parasite *Trypanosoma cruzi*, is a devastating vector-borne disease affecting six to seven million people worldwide. The disease is endemic in Latin American countries, but owing to globalized migration flows, it has lately become an emerging public health problem to nonendemic regions such as the U.S. and Europe.^1^ About 20–30% of infected individuals develop cardiomyopathy and/or digestive megasyndromes, leading to disability or death, and significant social and economic burden.^2^ The approved drugs for ChD treatment (i.e., benznidazole and nifurtimox) are very effective in the acute phase, which is rarely diagnosed in the majority of infected individuals. Chemotherapy in the chronic phase, however, is partially effective and may have serious side effects, resulting in premature termination of treatment in 10–20% of patients.^3^ It is estimated that no more than 1% of the chronic patients undergo treatment.^4^ There is no prophylactic or therapeutic vaccine for ChD.^5,6^

Over the years, many attempts have been made to develop experimental, preventive, and therapeutic vaccines using attenuated parasites, parasite lysates, or extracts; purified or recombinant protein subunits; and more recently, recombinant DNA. With few exceptions, however, most of these potential vaccine candidates provide partial to no protection against *T*. *cruzi* in different mouse models.^5,6^ A major bottleneck for the rational development of an effective protein- or peptide-based experimental vaccine to ChD is the limited proteomic information available on major strains, isolates, and clones representing the six parasite genotypes.^7–10^ This results in a scarcity of information on universal and conserved protein epitopes to be explored as experimental vaccine candidates. More recently, however, a recombinant adenovirus vaccine, using conserved gene sequences from the amastigote surface protein 2 (ASP2) and *trans*-sialidase (TS) family, has been evaluated as therapeutic vaccine candidate in mice, providing a significant reduction of cardiac pathology and improving disease outcome.^11^ In that study, the protective role of CD8^+^ T cells against experimental *T*. *cruzi* infection corroborated several previous observations (reviewed in ref. ^12^). Furthermore, a chimeric vaccine containing domains of ASP2 and TS, known as Traspain, also showed the ability to prime effector CD8^+^ T cells and control parasite dissemination.^13^ Other antigens such as Tc24 (or flagellar-calcium-binding protein) and trypomastigote surface antigen (TSA-1), both as recombinant proteins, also induced memory CD4^+^ and CD8^+^ T cells, resulting in parasite clearance, decrease of cardiac parasite burden, and long-term immunity.^14^ These vaccines and a few others^5,6^ are promising candidates; the conservation of these protein/peptide epitopes, however, among the six genotypes and their multitude of strains and isolates remains unproven. This is a recurrent issue in the development of effective peptide-/protein-based vaccines for ChD.

The *T*. *cruzi* glycocalyx is composed of abundant, complex, highly variable, and immunogenic glycosylphosphatidylinositol (GPI)-anchored glycoproteins and glycolipids, such as mucins, mucin-associated surface proteins (MASPs), TS/gp85 glycoproteins, and glycoinositolphospholipids.^10^ Different expression levels of these antigens are observed throughout the life-cycle stages of the parasite. For instance, in the infective host cell-derived trypomastigote (tissue culture-derived trypomastigote (TCT)) stage, the predominant glycoproteins belong to the mucin family, with members containing up to 60% of their molecular mass composed of *O*-glycans.^10,15,16^ Trypomastigote-derived GPI-anchored mucins (tGPI-mucins) contain the linear immunodominant glycotope Galα1,3Galβ1,4GlcNAc (Galα3LN) and several branched α-Gal-terminating *O*-glycans not yet fully characterized.^15^ These α-Gal glycotopes induce high levels of *T*. *cruzi-*specific anti-α-Gal antibodies in ChD patients (Ch anti-α-Gal Abs).^15,17–22^ The Ch anti-α-Gal Abs are protective, thus effectively controlling the parasitemia in both the acute and chronic phases of ChD and are universally and abundantly found in patients from endemic and nonendemic countries.^15,19–24^ Moreover, the Ch anti-α-Gal Abs have both specificity and biological activity considerably distinct from the so-called natural (or normal human serum (NHS)) anti-α-Gal Abs. The latter is elicited mainly against Gram-negative enterobacteria of the human gut flora,^25,26^ which express a wide variety of terminal nonreducing α-Gal-linked glycans, predominantly Galα1,2-, Galα1,4-, and Galα1,6-R, where R is the residual side chain or glycan on the lipopolysaccharide core oligosaccharides or *O*-antigens.^27^ Thus far, the immunodominant glycotope Galα3LN on tGPI-mucins, which is a major target for the Ch anti-α-Gal Abs, has not yet been reported in any enterobacteria.^27^ Therefore, in comparison with Ch anti-α-Gal Abs, the NHS anti-α-Gal Abs have a much weaker binding affinity to the linear Galα3LN^28,29^ glycotope and branched α-Gal-terminating *O*-glycans on tGPI-mucins.^15^ Consequently, the NHS anti-α-Gal Abs have substantially lower trypanolytic activity than Ch anti-α-Gal Abs on both TCTs and insect-derived metacyclic trypomastigotes.^15,19^

Since terminal, nonreducing, and linear α-Gal glycotopes (e.g., Galα1,3Gal, Galα1,3Galβ1,4GlcNAc) are absent in human tissues,^30^ we hypothesized that an effective vaccine against *T*. *cruzi* could be achieved using these highly immunogenic B cell glycotopes, in combination or not with CD8^+^ T cell epitopes. Actually, it has been recently suggested that α-Gal glycotope(s) could be employed in a pan-vaccine against major infectious diseases, such as malaria, ChD, leishmaniasis, African trypanosomiasis, and tuberculosis.^31^ In support of this idea, α-Gal-based vaccines have been shown to induce considerable protection against different species of *Plasmodium*^32^ and *Leishmania*.^33,34^ Thus far, however, no α-Gal-based vaccine has been tested in the context of experimental ChD.

Here we evaluated the trisaccharide Galα1,3Galβ1,4GlcNAc (Galα3LN), also known as the Galili or α-Gal epitope or glycotope,^26^ coupled to the carrier protein human serum albumin (Galα3LN-HSA), in the presence or not of adjuvant liposomal-monophosphoryl lipid A (LMPLA), as a potential experimental vaccine to acute ChD. To this end, we employed the α1,3-galactosyltransferase-knockout (α1,3GalT-KO) mouse model,^35,36^ which closely mimics human humoral responses against α-Gal glycotopes.

## Results

### α1,3GalT-KO mice are naturally more resistant to *T*. *cruzi* infection

**Fig. 1:**
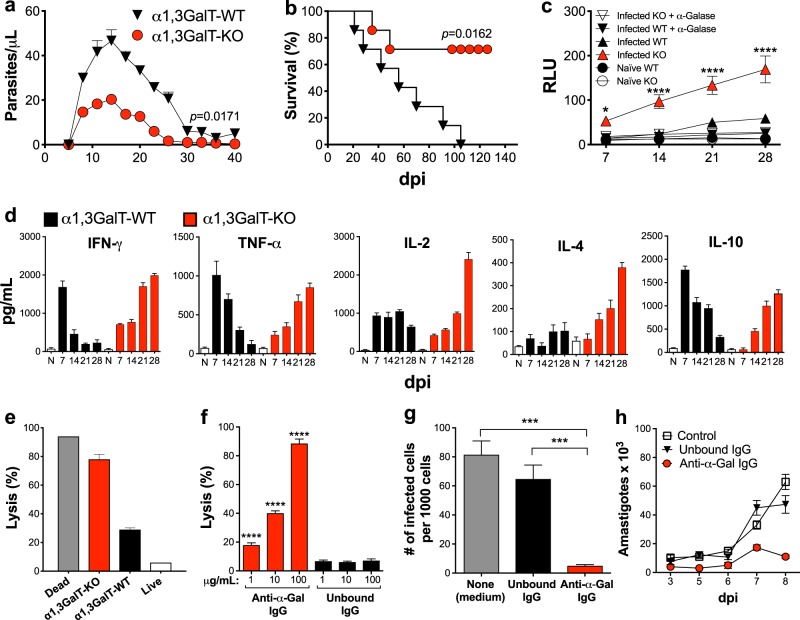
α1,3GalT-WT and α1,3GalT-KO mice exhibit different immune responses to *T*. *cruzi* infection. **a** Parasitemia in α1,3GalT-WT and α1,3GalT-KO mice infected (intraperitoneally (i.p.)) with 1 × 10^3^ tissue culture-derived trypomastigotes (TCTs) (Y strain). **b** Kaplan–Meier survival rate curve of animals infected with 1 × 10^4^ TCTs (Y strain). **c** Anti-α-Gal Ab levels as measured by chemiluminescent enzyme-linked immunosorbent assay using Galα3LN-BSA as antigen. RLU relative luminescence units. Infected C57BL/6 α1,3GalT-WT mice were compared to knockout (KO) mice by two-way analysis of variance (ANOVA) with Tukey’s multiple comparison test. *p < 0.05; ****p < 0.0001. **d** Serum cytokine profile of α1,3GalT-WT and α1,3GalT-KO mice infected (i.p.) with 1 × 10^3^ TCTs and followed up for 28 days. N naive. Lysis of TCTs (1 × 10^7^/mL) by serum **e** or purified anti-α-Gal Abs **f** from α1,3GalT-WT and α1,3GalT-KO mice infected (i.p.) with 1 × 10^3^ TCTs. Unbound immunoglobulin G (IgG), non-anti-α-Gal IgG antibodies (Abs) (flow through) from Synsorb 115 immunoaffinity chromatography; anti-α-Gal IgG, anti-α-Gal Abs eluted from Synsorb 115. Inhibition of parasite host cell invasion **g** and intracellular amastigote proliferation **h**. LLC-MK2 cells were infected with Y strain TCTs (multiplicity of infection = 10), for 2 h at 37 °C, in the presence or not of 100 μg/well of murine non-anti-α-Gal IgG Abs (Unbound IgG from Synsorb 115) or purified murine anti-α-Gal IgG Abs or control (None, medium alone). Number of infected cells per 1000 cells was evaluated after staining with 4,6-diamidino-2-phenylindole. **a**, **b**, **f**, **g** One-way ANOVA with Dunn’s multiple comparisons test. ***p < 0.001; ****p < 0.0001. The *p* values in **a**, **b** pertain to the mean values of the α1,3GalT-KO group in comparison with the α1,3GalT-WT throughout the course of the experiment. Error bars indicate S.E.M

Mice and all other mammals, except for humans and Old-World nonhuman primates, express the Galili or α-Gal glycotope on their cells and are therefore tolerant to this antigen.^26,30,37^ The α-Gal glycotope is the immunodominant epitope found in infective *T*. *cruzi* trypomastigote forms,^15,19^ responsible for eliciting high levels of protective anti-α-Gal Abs in both acute and chronic ChD.^17–20^ These Abs are capable of controlling the parasitemia in both disease stages in a complement-dependent and -independent manner.^15,19,20,22,38^ To substantiate the role of anti-α-Gal Abs in protection against the parasite in a well-defined experimental model, we employed the α1,3GalT-KO mouse model that, akin to humans, do not express terminal α-Gal glycotopes on their cells due to the disruption of the UDP-galactose:β-galactoside-α-1,3-galactosyltransferase (α1,3GalT) gene.^36,39^ First, we compared the response to *T*. *cruzi* infection of wild-type (α1,3GalT-WT) and α1,3GalT-KO mice, both in the C57BL/6 background. We infected (intraperitoneally (i.p.)) both groups of mice (*n* = 5–10, each) with 1 × 10^3^ TCTs of *T*. *cruzi* (Y strain). Mice were assessed for parasitemia, survival, and anti-α-Gal Ab titers. As observed in Fig. 1a, the acute parasitemia was much lower in α1,3GalT-KO mice than in the control WT group. The α1,3GalT-KO group also showed a much higher survival rate (~70%) in comparison to the WT group (Fig. 1b). In the latter, all mice died within 105 days postinfection (dpi). Next, we investigated whether the α1,3GalT-KO mice were able to produce specific anti-α-Gal Abs. For this, Galα3LN-BSA was used as antigen and chemiluminescent enzyme-linked immunosorbent assay (CL-ELISA)^29^ was performed as described in Methods. As expected, the α1,3GalT-KO group produced significantly higher levels of anti-α-Gal Abs during the course of infection when compared with the α1,3GalT-WT group (Fig. 1c). As expected, the α1,3GalT-WT group showed a much lower titer of anti-α-Gal Abs in total serum than the α1,3GalT-KO group. To assess the specificity of anti-α-Gal Abs produced by both groups, we treated the immobilized Galα3LN-BSA antigen with green coffee bean α-galactosidase to remove terminal α-Gal residues and observed no reactivity, suggesting that the majority of Abs were produced against the terminal nonreducing α-Gal glycotope. We then analyzed proinflammatory (interferon (IFN)-γ, tumor necrosis factor (TNF)-α, and interleukin (IL)-2) and regulatory (IL-4 and IL-10) cytokines in the serum of α1,3GalT-KO and -WT animals through the course of *T*. *cruzi* infection. α1,3GalT-WT mice showed high levels of IFN-γ, TNF-α, and IL-10 in the early stage of infection (day 7). However, these cytokines decreased significantly throughout the course of infection, reaching levels comparable to the naive (N) control mice at day 28. The levels of IL-2 and IL-4, on the other hand, slightly decreased or remained almost unaltered between 7 and 28 dpi in the α1,3GalT-WT mice (Fig. 1d). Conversely, α1,3GalT-KO mice showed increasingly higher levels of all cytokines tested from 7 to 28 dpi.

### Anti-α-Gal Abs from α1,3GalT-KO mice are protective as human anti-α-Gal Abs

When we expose TCTs to sera of 28 dpi from α1,3GalT-KO and -WT mice, high trypanolytic activity is observed, killing an average of 78% of TCTs after 1 h incubation at 37 °C (Fig. 1e). Similarly, *T*. *cruzi-*specific anti-α-Gal Abs purified from acute and chronic ChD patients have cytotoxic (trypanolytic) effects on both metacyclic trypomastigotes and TCT forms of the parasite in vitro.^19,20,22^ Next, we purified anti-α-Gal Abs produced by α1,3GalT-KO mice to investigate whether these Abs are also trypanolytic. First, the lytic property of the purified anti-α-Gal Abs was evaluated by incubation with TCTs at three different concentrations (1, 10, and 100 µg/mL), and motile parasites were counted. Unbound immunoglobulin G (IgG) (non-anti-α-Gal Abs) from the Galα3LN-Synsorb column was used as negative control (Fig. 1f). Purified anti-α-Gal Abs agglutinated and killed ~90% of the parasites (in a concentration-dependent manner) in the absence of complement, which is in agreement with the previous observations of anti-α-Gal Abs purified from acute or chronic ChD patients.^15,20,22^ To evaluate the ability of purified murine anti-α-Gal IgG Abs to prevent host cell infection and intracellular parasite proliferation, cells were infected and 100 μg/mL of murine anti-α-Gal Abs was added simultaneously. We observed that purified anti-α-Gal IgG Abs, but not unbound IgG Abs or medium alone, were able to significantly block infection (Fig. 1g). The number of intracellular amastigotes per cell (parasite proliferation), evaluated at 3–8 dpi, was significantly reduced, most likely due to the lower infection rate in the presence of purified murine anti-α-Gal IgG Abs but not of unbound IgG Abs or medium alone (negative control) (Fig. 1h). Taken together, these results clearly demonstrated that anti-α-Gal Abs elicited by *T*. *cruzi-*infected α1,3GalT-KO mice have cytotoxic and protective properties against the parasite similar to the anti-α-Gal Abs purified from ChD patients.^19,22^

### The α-Gal glycotope protects α1,3GalT-KO mice against *T*. *cruzi* infection

**Fig. 2:**
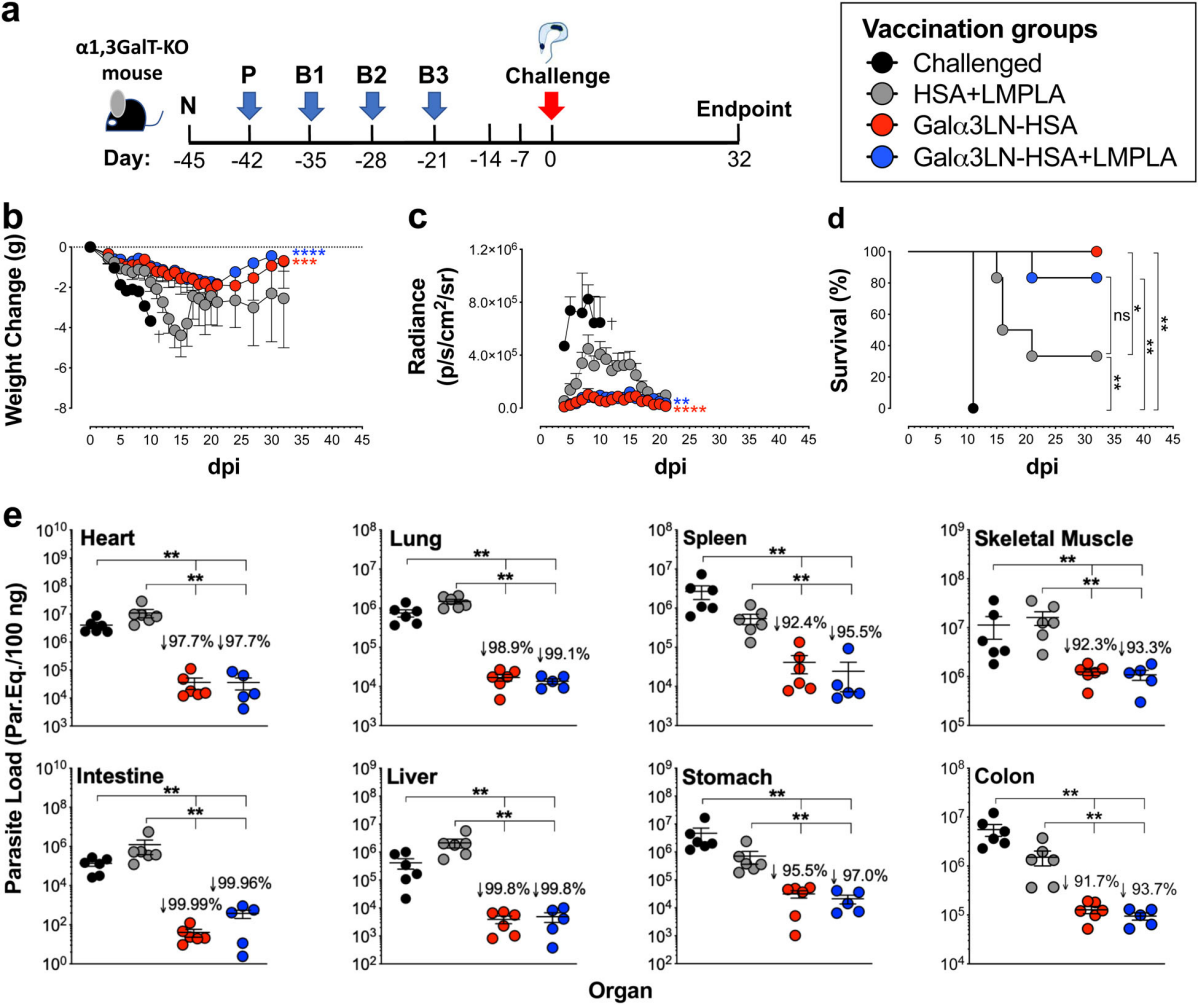
Vaccination with Galα3LN-HSA or Galα3LN-HSA+LMPLA protects α1,3GalT-KO mice against *T*. *cruzi* infection. **a** Experimental design scheme. N naive (before prime); P prime; B1–B3, boosts 1–3. Three weeks after B3, immunized α1,3GalT-KO mice were challenged (intraperitoneally) with 1 × 10^5^ *T*. *cruzi* CL Brener-*luc* tissue culture-derived trypomastigotes. Experimental endpoint was 32 days postinfection. **b** Weight change in grams (g) assessed in immunized (Galα3LN-HSA+/−LMPLA) and control (HSA+LMPLA) α1,3GalT-KO mice daily after parasite challenge. One-way analysis of variance (ANOVA) with Dunn’s multiple comparisons test was performed comparing the immunized (Galα3LN-HSA+/−LMPLA) groups with the control group (HSA+LMPLA); *p* values indicate the significance for the whole group. *p < 0.05; **p < 0.01; ***p < 0.001; ****p < 0.0001. Death of all animals (*n* = 3) in the challenged-only group is indicated (†). Error bars indicate S.E.M. **c** In vivo bioluminescence imaging of α1,3GalT-KO mice was performed to quantify parasite burden in the whole body following parasite challenge. Images were obtained daily and radiance (p/s/cm^2^/sr) was plotted. One-way ANOVA with Dunn’s multiple comparisons test was performed comparing the immunized (Galα3LN-HSA+/−LMPLA) groups with the control group (HSA+LMPLA); *p* values indicate the significance for the whole group. **p < 0.01; ****p < 0.0001. Error bars indicate S.E.M. Death of all animals (*n* = 3) in the challenged-only group is indicated (†). **d** Kaplan–Meier survival rate was assessed and recorded in each animal group (*n* = 6 per group in all groups, except for group “Challenged”) after parasite challenge. Log-rank (Mantel–Cox) test was performed to compare the immunized groups (Galα3LN-HSA+/−LMPLA) with the control groups (Challenged-only and HSA-LMPLA). *p < 0.05; **p < 0.01. **e** Determination of parasite load by quantitative real-time PCR in the heart, lung, spleen, skeletal muscle, intestine, liver, stomach, and colon, using satellite DNA of *T*. *cruzi*. Parasite load is expressed as parasite equivalents per 100 ng of tissue. Reduction of parasite load was calculated by dividing the mean of experimental vaccines by the HSA+LMPLA control group. Error bars indicate S.E.M. of triplicate determinations. Student’s *t* test with Mann–Whitney was performed to compare the immunized groups (Galα3LN-HSA+/−LMPLA) with the control groups (Challenged-only and HSA-LMPLA). **p < 0.01. The *p* values in **b**, **c** pertain to the mean values of each immunized group in comparison with the HSA+LMPLA control group throughout the course of the experiment

We evaluated whether immunization with a neoglycoprotein (NGP) containing the α-Gal glycotope could elicit protection against *T*. *cruzi* challenge. Groups of six female mice each were immunized with an NGP Galα3LN-HSA (20 μg per dose per mouse) alone or in combination with an adjuvant, LMPLA, Galα3LN-HSA+/−LMPLA. Control groups consisted of animals injected with the carrier protein and adjuvant combination (HSA+LMPLA), naive (nonimmunized, noninfected), or challenged (nonimmunized, infected) animals. The last two doses for all groups did not include LMPLA. As shown in Fig. 2a, serum was collected 3 days before prime immunization (naive, N) to obtain baseline levels. Then animal groups received a prime (P) and three boost immunizations (B1–B3), i.p., at 7-day intervals, followed by an i.p. *T*. *cruzi* challenge with 1 × 10^5^ TCTs (CL Brener clone) expressing red-shifted luciferase (CL Brener-*luc*),^40^ 3 weeks after the last immunization (B3). The experimental endpoint was 32 dpi, when all mice were humanely euthanized. Serum was obtained 3 days after each immunization and throughout the remainder of the experiment. Upon infection, mice were observed for abnormal behavior and toxicity signs, and their weights were recorded daily. Any weight change was normalized to the animal’s initial weight (Fig. 2b). We observed that both groups, Galα3LN-HSA (*p* < 0.001) and Galα3LN-HSA+LMPLA (*p* < 0.0001), lost less weight when compared to the control HSA+LMPLA group and started regaining weight few weeks postinfection. In contrast, animals in the HSA+LMPLA group lost considerable weight in the first 15 dpi, regaining some weight until the experimental endpoint.

The parasite burden in the three animal groups infected with TCTs of CL Brener-*luc* strain was then evaluated using an in vivo bioimager. To this end, mice were injected (i.p.) D-luciferin in sterile phosphate-buffered saline (PBS) and were anesthetized and imaged 10 min after substrate injection. Images of anesthetized mice were captured in automatic exposure and bioluminescence obtained was expressed in radiance (photons per second per square centimeter squared per steradian, p/s/cm^2^/sr) per animal (Fig. 2c). Upon challenge, the Galα3LN-HSA (*p* < 0.0001) and Galα3LN-HSA+LMPLA (*p* < 0.01) groups showed a total parasite burden significantly lower than the HSA+LMPLA control group. Moreover, both Galα3LN-HSA and Galα3LN-HSA+LMPLA exhibited a 100% and an 83% survival at endpoint, respectively, when compared to a 33% survival in the HSA+LMPLA control group (Fig. 2d).

To investigate whether the α-Gal glycotope-based vaccine could also reduce parasite burden, we performed quantitative real-time PCR (qPCR) analysis in the homogenized heart, lung, spleen, skeletal muscle, intestine, liver, stomach, and colon tissues (Fig. 2e). Animals vaccinated with Galα3LN-HSA+/−LMPLA showed a substantial decrease (91.7–99.9%) in parasite burden in all aforementioned tissues when compared to the HSA+LMPLA control group. Heart and skeletal muscle tissues of animals in the HSA+LMPLA control group showed a much higher parasite load (average of ~10^7^ parasite equivalents/100 ng tissue) than all other infected tissues (average of ~10^6^ parasite equivalents/100 ng tissue) in the same group. Moreover, the reduction in parasite load in all tissues was also highly significant when comparing both the immunized groups with the challenged only group. Taken together, our results indicated that animals vaccinated with Galα3LN-HSA, with or without adjuvant, have significantly reduced parasitic burden in all tissues analyzed. Taken together, our data clearly demonstrate that immunization of α1,3GalT-KO mice with an NGP containing the α-Gal glycotope, in the presence or not of an adjuvant, can significantly protect mice against lethal parasite challenge.

### Kinetics and isotype profile of humoral response in α1,3GalT-KO mice following Galα3LN-HSA or Galα3LN-HSA+LMPLA vaccination

**Fig. 3:**
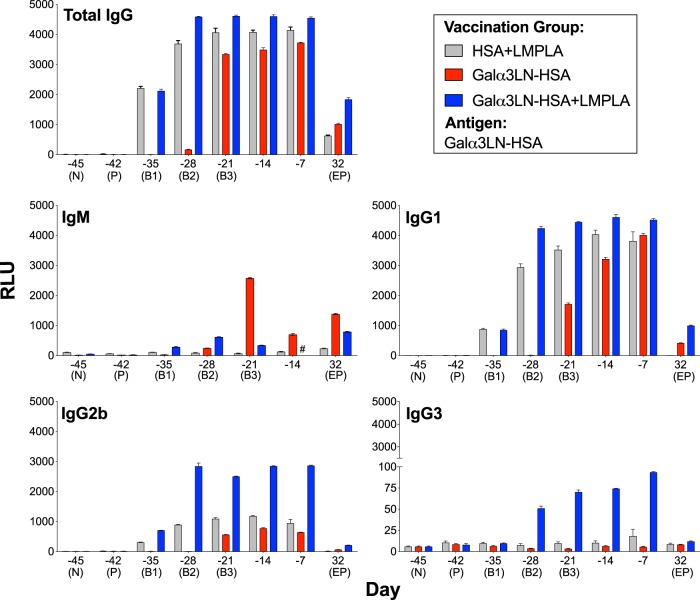
Kinetics of humoral immune response elicited by Galα3LN-HSA, Galα3LN-HSA+LMPLA, and HSA+LMPLA. Chemiluminescent enzyme-linked immunosorbent assay (CL-ELISA) reactivity of mouse sera from α1,3GalT-KO mice injected with Galα3LN-HSA, Galα3LN-HSA+LMPLA, or HSA+LMPLA using Galα3LN-HSA as immobilized antigen. For immunoglobulin M (IgM) measurement, prior to the CL-ELISA we depleted IgG antibodies by preincubating each serum sample with a 15-μL bead suspension mixture of protein A-Sepharose 4B and protein G-Sepharose, both fast flow (A:G, 1:1, v/v), and recombinant human serum albumin (HSA; 1 mg/mL), for 1 h at 37 °C on rotatory shaker. Error bars indicate S.E.M. of triplicate determinations. Sample no longer available is indicated (#). RLU relative luminescence units

To assess the development of a humoral immune response, sera were collected from the vaccinated and control groups 3 days after each timepoint (Fig. 2a), and the kinetics of specific Ab isotypes (IgG, IgM, IgG1, IgG2b, IgG3, IgA, and IgE) were evaluated (Fig. 3 and Supplementary Figure 1). As anticipated, an early (B1 and B2) and higher total IgG response was observed in the animals vaccinated with Galα3LN-HSA plus adjuvant (Galα3LN-HSA+LMPLA) when compared with mice immunized with Galα3LN-HSA alone (Fig. 3). In contrast, a lower IgM anti-α-Gal response was observed mainly in the Galα3LN-HSA group, following IgG depletion using protein A-/G-Sepharose. Furthermore, IgG1 was the main IgG isotype, followed by substantial levels of IgG2b elicited in both the α-Gal-vaccinated groups and HSA+LMPLA control (Fig. 3). Low levels of IgG3 were detected only in the Galα3LN-HSA+LMPLA group. Following parasite challenge, α-Gal-vaccinated mice continued to exhibit higher amounts of IgG1 and IgG2b Abs when compared with the baseline (N, day −45) levels. However, these isotypes were considerably reduced at the endpoint. For example, for total IgG, a reduction to 27% and 40% of the total maximum reactivity was observed for the Galα3LN-HSA and Galα3LN-HSA+LMPLA groups at day −7, respectively (Fig. 3, total IgG). However, this reduction cannot be directly correlated to Ab functionality (i.e., lytic activity) in vivo or in vitro. We estimated the concentration of anti-α-Gal Abs at day 32 dpi to be 83 and 214 μg/mL for the Galα3LN-HSA and Galα3LN-HSA+LMPLA groups, respectively. Although reduced, these Ab concentrations are still high and sufficient to effectively kill the parasites (see section below).

IgA and IgE responses, on the other hand, were virtually nonexistent following vaccination with Galα3LN-HSA+/−LMPLA (Supplementary Figure 1). We also observed a very strong response to Galα3LN-HSA of total IgG, IgG1, and IgG2b in the control group injected with HSA+LMPLA. This response was significantly reduced or absent at the endpoint as well (Fig. 3). We hypothesized that this strong background response could be due to Abs against HSA. Aiming to address this background reactivity, we used different proteins as immobilized antigens in the CL-ELISA to measure the levels of non-anti-α-Gal Abs elicited in the Galα3LN-HSA-immunized group during different timepoints (Supplementary Figure 2). We observed an increasing reactivity to HSA following boosts 2 and 3 (6% and 15% of the total reactivity), reaching a plateau at day −14 (35%). Furthermore, we could not detect any cross-reactivity to other proteins (i.e., bovine serum albumin (BSA) and casein), used as immobilized antigen (Supplementary Figure 2). In summary, Galα3LN-HSA, Galα3LN-HSA+LMPLA, and HSA+LMPLA induced a strong humoral response with the prevalence of IgG1 and IgG2b isotypes.

### Antibodies elicited by α-Gal-based vaccine recognize terminal α-Gal residues, have trypanolytic activity, and bind to specific α-Gal-containing domains on the parasite surface

**Fig. 4:**
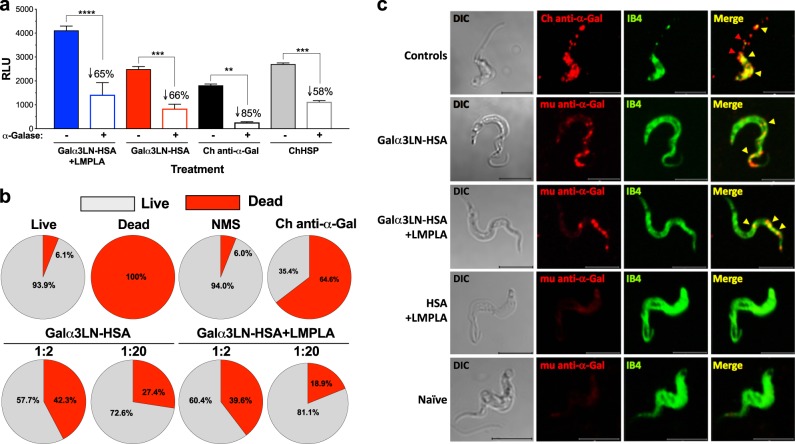
Anti-Galα3LN-HSA Abs are α-Gal-specific, have lytic activity, and recognize parasite surface glycotopes. **a** Chemiluminescent enzyme-linked immunosorbent assay reactivity of sera from α1,3GalT-KO mice vaccinated with Galα3LN-HSA+/−LMPLA before and after green coffee bean α-galactosidase (α-Galase) treatment of the immobilized Galα3LN-HSA antigen. Purified human Chagas (Ch) anti-α-Gal immunoglobulin G (IgG) antibodies (Abs) and human serum pool from chronic Ch disease patients (ChHSP) were used as controls. RLU relative luminescence units. One-way ANOVA with Sidak’s multiple comparison test was performed comparing groups before (−) and after (+) α-galactosidase (α-Galase) treatment; *p* values indicate the significance of group before and after treatment. **p < 0.01; ***p < 0.001; ****p < 0.0001. **b** Percentage of lysis of tissue culture-derived trypomastigotes (TCTs) incubated with sera from α1,3GalT-KO mice vaccinated with Galα3LN-HSA+/−LMPLA. **c** Confocal microscopy using murine (mu) anti-α-Gal IgG Abs purified from mice vaccinated with Galα3LN-HSA+/−LMPLA. *Bandeiraea simplicifolia* Isolectin IB4 was used to label all terminal α-Gal residues on TCT surface. Human Ch anti-α-Gal IgG Abs were used as positive control. Bar 5 μm

To determine the specificity of the Abs produced against Galα3LN-HSA+/−LMPLA, microplate-immobilized Galα3LN-HSA was treated with α-galactosidase to remove terminal α-Gal residues, and the serum reactivity from mice immunized with Galα3LN-HSA or Galα3LN-HSA+LMPLA, was determined by CL-ELISA,^33^ as detailed in Methods (Fig. 4a). A significant decrease in the reactivity of sera from both Galα3LN-HSA (*p* < 0.001, 66% reduction) and Galα3LN-HSA+LMPLA (*p* < 0.0001, 65% reduction) group was observed. The remaining reactivity was confirmed to be against HSA (Supplementary Figure 2). As positive controls, purified anti-α-Gal Abs (Ch anti-α-Gal Abs) and a pool of sera (ChHSP), both from chronic ChD patients, were used. Significant decrease in Ab reactivity to the α-galactosidase-treated antigen was also observed with Ch anti-α-Gal Abs (85%) and ChHSP (58%) (Fig. 4a).

To address whether the anti-α-Gal Abs elicited by immunization with Galα3LN-HSA+/−LMPLA are trypanolytic, sera from both animal groups were incubated with TCTs for 30 min at 37 °C, without complement, and live and dead parasites were manually counted (Fig. 4b). In the Galα3LN-HSA-vaccinated group, we observed 42.3% (±3.9) and 27.4% (±1.0) parasite deaths at 1:2 and 1:20 serum dilutions, respectively. Likewise, in the Galα3LN-HSA+LMPLA-immunized group, we observed 39.6% (±3.13) and 18.9% (±3.1) parasite deaths at 1:2 and 1:20 dilutions, respectively, suggesting a dose-dependent effect. A naive mouse serum (NMS) was used as negative control and a low parasite death (6.0%, ±0.1), not significantly different from that of the live parasite control (medium alone) (6.1%, ±1.0), was observed. This result was further corroborated by the very low binding to the parasite surface of anti-α-Gal Abs purified from mice immunized with HSA+LMPLA (Fig. 4c). As positive controls, parasites were incubated with 4% formaldehyde or Ch anti-α-Gal Abs, resulting in 100% and 64.6% (±0.74) parasite deaths, respectively (Fig. 4b). It is important to point out that the Ch anti-α-Gal Ab-mediated trypomastigote lysis occurred in the absence of complement, corroborating therefore previous observations.^22^ Similarly, the parasite lysis caused by antiserum from mice immunized with Galα3LN-HSA+/−LMPLA was complement independent.

To assess whether anti-α-Gal Abs elicited by immunization with Galα3LN-HSA+/−LMPLA recognize α-Gal-rich domains on the TCT surface, we performed immunoaffinity purification of these Abs using nitrocellulose-immobilized Galα3LN-HSA, followed by confocal microscopy. The murine (mu) anti-α-Gal IgG Abs from animals immunized with Galα3LN-HSA+/−LMPLA strongly recognize speckled regions toward the parasite’s anterior end and mostly on the flagellar membrane (Fig. 4c, second and third panel rows from the top, yellow arrowheads). These domains partially colocalize with *Bandeiraea* (*Griffonia*) *simplicifolia* isolectin IB4, which has a broad specificity toward terminal nonreducing α-Gal residues^41^ (Fig. 4c, second and third row from the top, last panel, yellow arrowheads). Conversely, human Ch anti-α-Gal Abs exhibited an extensive colocalization with IB4 lectin (Fig. 4c, Controls, fourth panel, yellow arrowheads), strongly indicating that both are recognizing a more diverse array of α-Gal glycotopes on the parasite surface as compared to the murine anti-α-Gal Abs. Some areas on the parasite surface located toward the flagellum, however, did not have any colocalization with IB4 (Fig. 4c, Controls, fourth panel, red arrowheads). As negative controls, we used natural anti-α-Gal Abs immunopurified from HSA+LMPLA-immunized or naive animals. These natural anti-α-Gal Abs showed very low binding to the parasite-specific α-Gal glycotopes (Fig. 4c, bottom rows).

### The α-Gal vaccine in combination with the adjuvant LMPLA induce upregulation of cytokines and chemokines following vaccination and *T*. *cruzi* infection

The production of different cytokines and chemokines is essential for a protective cell-mediated immune response against *T*. *cruzi* infection.^42^ Thus we evaluated whether immunization with Galα3LN-HSA+/−LMPLA or HSA+LMPLA could elicit a cell-mediated immune response, by using a bead-based Multiplex assay. First, a panel of 32 mouse cytokines, chemokines, and growth factors were analyzed in sera from immunized and *T*. *cruzi-*challenged mice at the experimental endpoint (32 dpi). In comparison to the challenged group, only the group of mice vaccinated with Galα3LN-HSA in combination with the adjuvant LMPLA displayed significantly higher levels of serum cytokines, particularly, IL-2, IL-4, IL-9, IL-15 (1.3–3.1-fold change), chemokine C-C chemokine motif ligand 3 (CCL3; 1.5-fold change), and growth factor vascular endothelial growth factor (VEGF; 4-fold change) (Supplementary Table 1). Furthermore, we observed no significant upregulation of these molecules in the Galα3LN-HSA- or HSA+LMPLA-immunized groups. These results suggest that the α-Gal-vaccine, in the presence of the adjuvant LMPLA, could induce the secretion of these cytokines, chemokines, and growth factor, following immunization and acute *T*. *cruzi* infection.

### Galα3LN-HSA vaccination elicits antigen-specific CD4^+^ T cell response

**Fig. 5:**
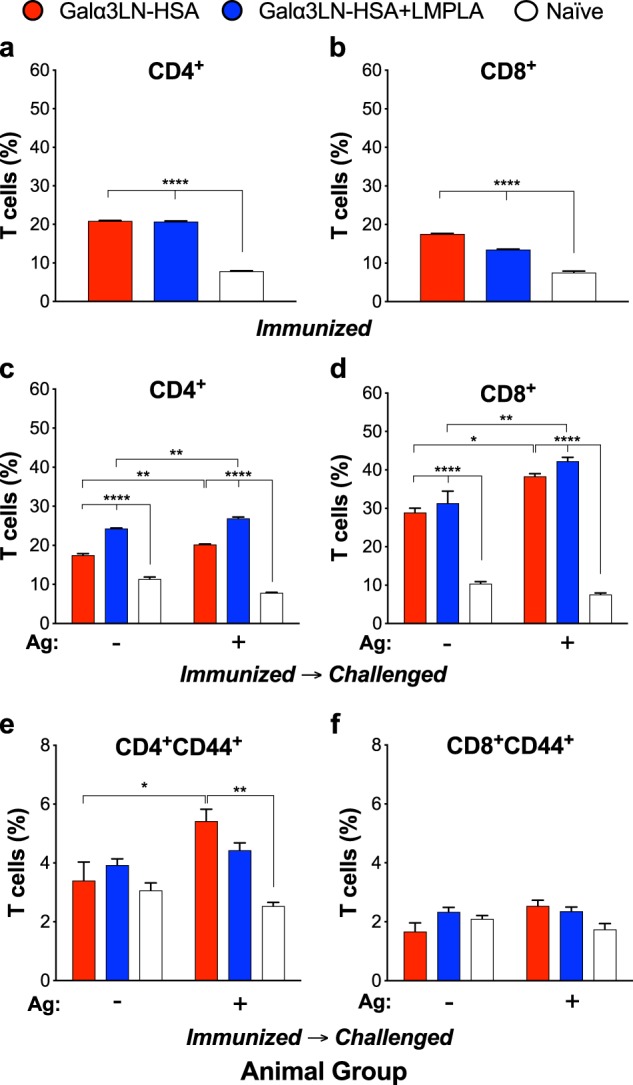
Vaccination with Galα3LN-HSA+/−LMPLA induce T cell activation. Splenocytes were collected from mice immunized with Galα3LN-HSA or Galα3LN-HSA+LMPLA and immunized-challenged mice, followed by ex vivo stimulation with 20 μg/mL Galα3LN-HSA. **a**, **b** Percentage of antigen-specific CD4^+^ and CD8^+^ T cells, respectively, in immunized mice. **c**, **d** Percentage of CD4^+^ and CD8^+^ T cells, respectively, in immunized-challenged mice. **e**, **f** Percentage of memory CD4^+^CD44^+^ and CD8^+^CD44^+^ T cells, respectively, in immunized-challenged mice. One-way analysis of variance (ANOVA) compared with naive group was obtained for the immunized groups; Two-way ANOVA compared with the naive group was obtained for the immunized-challenged groups; *p < 0.05; **p < 0.01; ****p < 0.0001. Error bars indicate S.E.M. of triplicate determinations

Next, we investigated whether Galα3LN-HSA+/−LMPLA could induce antigen-specific CD4^+^ and CD8^+^ T cells. To this end, splenocytes from α1,3GalT-KO mice vaccinated with Galα3LN-HSA, Galα3LN-HSA+LMPLA, or naive control group were stimulated in vitro with 20 μg/mL of Galα3LN-HSA antigen. We found a very significant (*p* ≤ 0.0001) increase of antigen-induced CD4^+^ and CD8^+^ T cells in both immunized (Fig. 5a, b), and immunized-challenged mice (Fig. 5c, d) but not in the naive control group. Next, we investigated whether antigen-specific CD4^+^ and CD8^+^ T cells differentiated into memory CD44^+^ T cells in α-Gal-immunized and parasite*-*challenged mice. Galα3LN-HSA but not Galα3LN-HSA+LMPLA generated a small but significant expansion of antigen-specific memory CD4^+^CD44^+^ T cells (Fig. 5e). On the other hand, no significant increase of memory CD8^+^CD44^+^ T cells were observed with either vaccine (Fig. 5f). Taken together, these results indicate the ability of the α-Gal-based vaccine alone to generate antigen-specific memory CD4^+^ T cells, critical for an effective protective anti-α-Gal IgG Ab immunity against *T*. *cruzi* infection.^15,19^

### α-Gal-vaccinated animals have significantly decreased cardiac inflammation; myocyte necrosis; and CD3^+^, CD4^+^, and CD8^+^ T cell infiltration

**Fig. 6:**
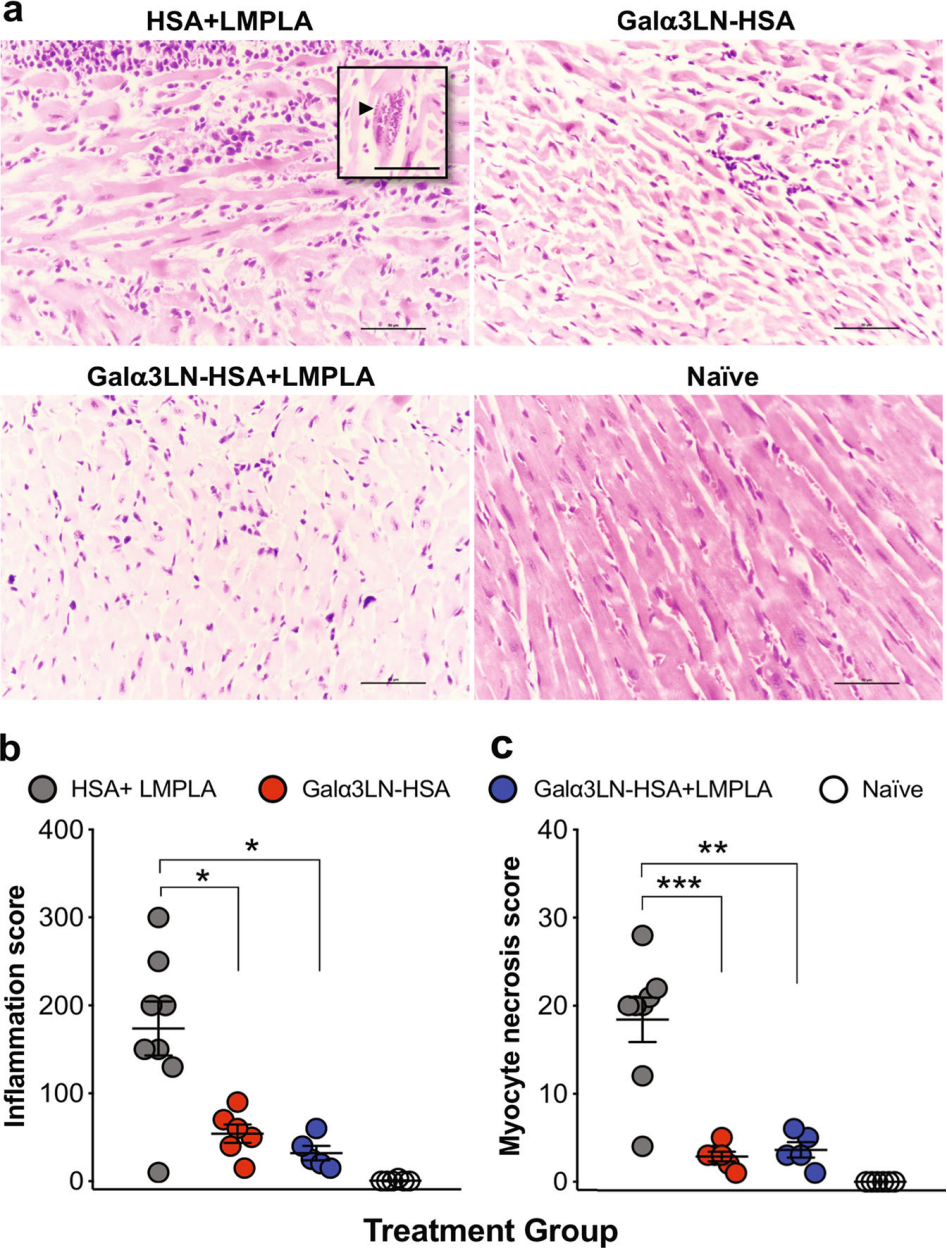
Histopathology analysis. **a** Micrographs of heart sections harvested at the endpoint and stained with hematoxylin and eosin. Inset, arrowhead indicates an amastigote nest. **b** Myocardial inflammatory and mononuclear cell infiltrates were manually scored. **c** Myocyte necrosis was manually counted in 25 fields (×400). Magnification ×40, bar 100 μm. Statistical significance was calculated by unpaired two-tailed Mann–Whitney test, comparing Galα3LN-HSA+/−LMPLA group to HSA+LMPLA group. *p* values indicate the significance *p < 0.05; **p < 0.01; ***p < 0.001

At the experimental endpoint (32 dpi), we also performed histopathological analysis of cardiac inflammation and myocyte necrosis in the three immunized and challenged groups. The number of mononuclear cells, including lymphocytes and macrophages, was counted in the ventricle sections. The necrosis of individual myocytes was also manually quantified. Figure 6a shows representative micrographs of heart sections of animals from the HSA+LMPLA, Galα3LN-HSA, or Galα3LN-HSA+LMPLA groups. Clearly, animals in the HSA+LMPLA group exhibited much higher cardiac inflammation and myocyte necrosis than animals vaccinated with Galα3LN-HSA+/−LMPLA. Moreover, amastigote nests were observed in most animals of the HSA+LMPLA group but not in any animal vaccinated with Galα3LN-HSA+/−LMPLA (Fig. 6a, inset). Quantification of the cardiac inflammation and myocyte necrosis shows significant differences between animals of the HSA+LMPLA group and those vaccinated with Galα3LN-HSA+/−LMPLA (Fig. 6b). However, no significant differences were observed between the two latter groups, indicating that LMPLA adjuvant did not provide any additional protection. Moreover, heart sections from animals immunized with Galα3LN-HSA+/−LMPLA or HSA-LMPLA control, subsequently challenged with *T*. *cruzi*, were also evaluated for infiltration of CD3^+^, CD4^+^, and CD8^+^ T cells at the experimental endpoint. Corroborating with the inflammation data (Fig. 6), α1,3GalT-KO mice immunized with the α-Gal-based vaccine, in the presence or not of an adjuvant, exhibited a much lower number of infiltrating CD3^+^, CD4^+^, and CD8^+^ T cells (Supplementary Figure 4).

## Discussion

As globalization increases, so have the reported cases of individuals infected with *T*. *cruzi*, with a rising number living in nonendemic regions such as the U.S. and Europe.^43,44^ Despite considerable efforts to control insect vectors^45^ and development of more effective chemotherapeutics^3^ and diagnostics,^46,47^ an effective prevention of ChD remains a challenge worldwide. Thus a prophylactic ChD vaccine is of paramount necessity considering that an estimated 25 million people are at risk of contracting the disease just in Latin America^48^ and many thousands more in nonendemic areas, including several regions in the U.S. and Europe.^44,49^

In recent years, several experimental vaccines based primarily on *T*. *cruzi* recombinant proteins and peptides and DNA have been developed.^6,12,50^ Although a few of these vaccines are highly effective in protecting mice against laboratorial parasite strains, there is no evidence so far that their target epitopes are conserved among the six parasite genotypes and a multitude of field isolates.^9^ Thus far, none of these vaccines have moved to clinical trials. One key obstacle is the extensive diversity of major parasite surface proteins, encoded mainly by three multigene families (i.e., mucins, TSs, and MASPs).^10,51–53^ The highly variable protein coat, owing to the simultaneous and differential expression of hundreds of sequence-coding genes in the two mammal-dwelling parasite stages and different strains^10,51–53^, hinders the development of an effective and universal vaccine targeting major parasite proteins.

Glycoconjugates, on the other hand, have recently become increasingly attractive molecular targets against trypanosomatids such as *T*. *cruzi* and *Leishmania* spp., both as biomarkers and potential vaccine candidates.^24,29,33,34,55,56^ In *T*. *cruzi*, its complex carbohydrate-rich surface coat contains GPI-anchored glycoproteins such as mucins, which exhibit the immunodominant glycotope Galα1,3Galβ1,4GlcNAc, linked mostly to a threonine residue.^15^ This highly immunogenic glycotope elicits lytic and protective anti-α-Gal Abs, which in turn controls the parasitemia in both the acute and chronic phases of the disease.^15,18–21^ With this in mind, we have used the linear trisaccharide, bound to human albumin, Galα3LN-HSA, which is almost identical to the native glycotope found on tGPI-mucins, except for the GlcNAc anomeric configuration, which is α on the latter. To mimic a human response, the efficacy of the α-Gal-containing vaccine (Galα3LN-HSA) was evaluated in the α1,3GalT-KO mouse model, which, in comparison to WT mice, does not express terminal, linear α-Gal glycotopes.^35,36^ As a result, α1,3GalT-KO mice produce high levels of anti-α-Gal Abs when infected with *T*. *cruzi*.^57^

First, we investigated the immune response differences between the C57BL/6 α1,3GalT-KO and WT mice infected with *T*. *cruzi*, keeping in mind the similarities that the former have with humans and Old-World nonhuman primates in that they elicit high-affinity Abs to the linear α-Gal glycotope. We observed that α1,3GalT-WT mice were unable to produce a robust immune response against *T*. *cruzi* via generation of protective anti-α-Gal Abs and a balanced serum cytokine profile, severely affecting their survival rate. In contrast, the α1,3GalT-KO animals could more effectively control the infection by decreasing parasitemia, likely due to the production of high levels of anti-α-Gal Abs and a balanced T helper type 1 (Th1)/Th2 cytokine response. We speculate that the latter could be explained by a mechanism of anti-α-Gal-mediated antigen-presenting cell (APC) targeting, as proposed by Uri Galili and collaborators.^58–60^ For instance, these authors demonstrated that IgG anti-α-Gal Abs produced against α-Gal-glycolipids loaded onto the surface of liposomes containing encapsulated chicken ovalbumin (OVA) could opsonize these antigens via Fc-gamma receptor (FcγR) present on the surface of APCs such as dendritic cells, leading to improved transport, processing, and presentation of the immunodominant OVA-derived SIINFEKL peptide to CD8^+^ T cells. This resulted in an increased immunogenicity of the vaccine and induction of IFN-γ by both CD8^+^ and CD4^+^ T cells.^59^ The same mechanism was observed by intradermal injection of α-Gal-containing nanoparticles in the skin of α1,3-GalT-KO mice, resulting in the upregulation of various cytokine and chemokine genes, including VEGF, fibroblast growth factor, IL-1, platelet-derived growth factor, and colony-stimulating factor (CSF).^60^ In our current study, the induction of anti-α-Gal Abs by TCTs in α1,3-GalT-KO mice, but not in α1,3-GalT-WT animals, could have resulted in improved presentation of parasite-derived peptides to CD4^+^ and CD8^+^ T cells, ensuing the production of Th1 and Th2 cytokines required for an effective protective response against the parasite.

Furthermore, the anti-α-Gal Abs, purified from α1,3GalT-KO mice, were lytic to TCTs in a complement-independent manner, as previously demonstrated for human Ch anti-α-Gal Abs.^22^ We have also shown here that purified murine anti-α-Gal IgG Abs could effectively block host cell infection and intracellular proliferation of the parasite. In sum, α1,3GalT-KO and α1,3GalT-WT mice clearly demonstrated an opposite behavior regarding the development of the infection and immune response in the acute phase of ChD. Our data support previous findings that acutely *T*. *cruzi*-infected α1,3GalT-KO mice exhibit lower parasitemia and increased production of anti-α-Gal Abs,^57^ which can be key to eliminate circulating parasites and control the disease.^26,61^

Since α1,3GalT-WT demonstrated to be a poor model to test protection against the heavily glycosylated *T*. *cruzi* TCT forms, we proceeded to test our α-Gal-based vaccine candidate (Galα3LN-HSA+/−LMPLA) in the α1,3GalT-KO mouse model. Evidently, high protection was observed at early infection stages, the parasite burden was drastically reduced, while the survival rate and animal weight were stable throughout the course of the experiment. To corroborate these results, the parasite systemic dissemination on selected individual organs was analyzed by qPCR. Interestingly, *T*. *cruzi* CL Brener-*luc* strain is not only myotropic but also localize to a number of tissues, particularly colon and stomach.^40,62^ Here we observe a decrease in parasitic burden >90% in all harvested tissues (i.e., heart, lung, spleen, skeletal muscle, intestine, liver, stomach, colon) from either Galα3LN-HSA- or Galα3LN-HSA+LMPLA-vaccinated mice when compared with the HSA+LMPLA control group.

Next, we evaluated the humoral immune response in α1,3GalT-KO mice immunized with Galα3LN-HSA, Galα3LN-HSA+LMPLA, or HSA+LMPLA. Both αGal-vaccinated groups elicited more IgG1 and IgG2b than IgG3 Abs. Our results are similar to those from a recent study using C57BL/6 α1,3GalT-KO mice immunized with the NGP Galα3LN-BSA, in which high levels of IgG1, IgG2b, and IgG3 Abs were observed.^32^ Previous studies have shown that IgG1 and IgG2b are associated with protection against *T*. *cruzi* infection.^63,64^ Furthermore, in an effort to improve the humoral immune response, Galα3LN-HSA was formulated with the LMPLA adjuvant.^65^ It is recognized that LMPLA acts inducing a Th1-biased immune and Ab responses, rich in IgG1 and IgG3 isotypes in humans,^66^ and preferentially IgG2a in mice.^67^ These Igs have the ability to opsonize and destroy infected cells and enhance the production of CTL CD8^+^ T cells (reviewed in ref. ^66^). Here the addition of LMPLA adjuvant significantly increased the levels IgG1, IgG2b, and IgG3 isotypes compared to Galα3LN-HSA alone, in which no significant levels of IgG3 were detected. Since C57BL/6 mice do not express IgG2a due to a genetic mutation,^68^ this immunoglobulin was not measured in our experiments.

Furthermore, we could not detect a significant production of IgA in either the Galα3LN-HSA- or the Galα3LN-HSA+MPLA-vaccinated group, even after depletion of IgG Abs that could compete for the binding to the immobilized Galα3LN-HSA antigen on CL-ELISA. This result corroborates the previous observation by LaTemple and Galili^69^ that IgA Abs are not elicited following immunization of C57BL/6 α1,3GalT-KO mice with an α-Gal-containing antigen. Moreover, in our study, we also could not detect any IgE Abs in the α-Gal-vaccinated groups, even following depletion of IgG Abs. The absence of IgE Abs is noteworthy, because it minimizes concerns about induction of autoimmune- or allergy-related IgE following vaccination with either α-Gal-vaccine formulation tested here. IgE anti-α-Gal Abs are linked to anaphylactic reactions to non-primate mammalian meat and other types of food^70^ and to recombinant monoclonal Abs (e.g., Cetuximab).^71^ In this case, the α-Gal glycotopes are in complex-type *N*-glycans containing terminal nonreducing α-Gal residues 1,3-linked to *N*-acetyllactosamine (Galα1,3Galβ1,4GlcNAcβ-R). By contrast, in *T*. *cruzi* tGPI-mucins the α-Gal glycotopes are found in the linear *O*-glycan trisaccharide, Galα1,3Galβ1,4GlcNAcα, or in as-yet uncharacterized branched *O*-glycans, both directly linked to threonine or serine residues.^15^ In this study, the Galα3LN glycotope is covalently linked to HSA via a 3-atom spacer, thus more closely resembling a short *O-*glycopeptide than a long complex-type *N*-glycopeptide. We speculate that the differential presentation of α-Gal-containing *N*- and *O*-glycopeptides by activated B cells via major histocompatibility complex (MHC) class II to CD4^+^ T cells could lead to distinct isotype switching (IgM to IgG or IgM to IgE).

The specificity and lytic power of murine anti-α-Gal Abs being produced upon vaccination with Galα3LN-HSA+/−LMPLA was further explored by treating the target glycotope with α-galactosidase. With the removal of terminal α-Gal residues, the reactivity in CL-ELISA of the serum from mice immunized with Galα3LN-HSA+/−LMPLA significantly decreases (~65%). The residual reactivity could be due to Abs to the HSA moiety, which was corroborated by the strong total IgG reactivity observed in animals immunized with HSA+LMPLA. Moreover, sera from mice immunized with either vaccine formulation killed the parasites in 30 min, in a dose-dependent manner, in the absence of complement. This corroborates the idea that binding of Ch anti-α-Gal Abs to the parasites destabilizes the plasma membrane by clustering abundant α-Gal-containing glycoproteins, such as tGPI-mucins, and disrupt the surface coat, causing blebs, agglutination and, ultimately, death.^22^ This mechanism has been well established in *T*. *cruzi* TCTs and, more recently, has also been proposed in *Leishmania major*.^33^ The labeling of TCTs with purified murine anti-α-Gal Abs is predominantly located to the parasite flagellum and resembles a “patchwork quilt” as recently observed by Lantos et al.^72^ A similar but more extensive labeling pattern was observed with purified Ch anti-α-Gal Abs, indicating that other α-Gal specificities are present in chronic ChD. Indeed, besides the linear Galα1,3Galβ1,4GlcNAcα trisaccharide, tGPI-mucins encompass several as-yet uncharacterized α-Gal-containing branched *O-*glycans, which are also strongly recognized by Ch anti-α-Gal Abs.^15^ IB4-Lectin has broad specificity toward terminal nonreducing α-Gal residues, primarily recognizing Galα1,3- and, to a lesser extent, Galα1,6-containing oligosaccharides, although other α-Gal linkages should be considered.^41^ This might explain the more extensive and homogenous recognition of parasite membrane by this lectin than Galα3LN-HSA-specific anti-α-Gal Abs.

In this study, at 32 dpi, we observed a significant upregulation of IL-2, IL-4, IL-9, IL-15, CCL3, and VEGF in the serum of α1,3GalT-KO mice immunized with Galα3LN-HSA only in the presence of the adjuvant LMPLA. Interestingly, no significant increase was exhibited in the Galα3LN-HSA or HSA+LMPLA immunized–challenged groups. Cytokines and chemokines play an important role in the protective immune response generated against *T*. *cruzi* infection.^73^ For instance, co-administration of a plasmid DNA encoding murine IL-15 together with *T. cruzi* TS DNA vaccine increased long-term protection against *T*. *cruzi* lethal infection.^74^ IL-15 has also been associated with survival and proliferation of memory CD4^+^ and CD8^+^ T cells.^74,75^ In addition, CC chemokine ligands CCL3 enhance the uptake of *T*. *cruzi* by macrophages, promoting nitric oxide that exerts trypanocidal activity, regulating parasite replication.^76^ Furthermore, VEGFβ, a family member of VEGF, has been demonstrated to be important against heart failure.^77^ IL-2 and IL-4 are known to promote the generation of IL-9 cytokine.^78^ However, the role of IL-9 in *T*. *cruzi* or ChD has not been studied. Altogether, these results indicate that the cytokine and chemokine response could be elicited by the α-Gal vaccine plus the adjuvant LMPLA, which is a strong activator of Toll-like receptor 4-mediated signaling pathway^65,79^ and not by the carrier protein HSA or adjuvant alone, thus modulating an effective protective host immune defense against infection. However, we do not discard the possibility that this combination may lead to excessive inflammation, affecting the survival of Galα3LN-HSA+LMPLA immunized mice, as observed here.

Previous studies showed that mice deficient in the MHC (MHC I and MHC II)^80^ or in both CD4^+^ and CD8^+^ T cells^81,82^ suffered high mortality and increased parasitemia following *T*. *cruzi* infection. Furthermore, during parasite infection, CD4^+^ T cells are capable of secreting Th1 cytokines (e.g., IFN-γ), which are crucial for macrophage activation and subsequent inhibition of the parasite. These cytokines can also induce the expansion of cytotoxic CD8^+^ T cells, which destroy infected cells.^42^ Therefore, the production of CD4^+^ and CD8^+^ T cell-mediated responses is considered to be essential for the control and protection against *T*. *cruzi* infection.^12,83^ Here we evaluated whether antigen-specific CD4^+^ and CD8^+^ T cells were induced by Galα3LN-HSA+/−LMPLA vaccination before and after *T*. *cruzi* challenge. We observed a statistically significant cell-mediated immune response led by the production of CD4^+^ and CD8^+^ T cells before and after parasite infection. Moreover, following stimulation with Galα3LN-HSA, we could observe a small but significant presence of CD4^+^/CD44^+^ but not of CD8^+^/CD44^+^ T lymphocytes in the spleen of animals vaccinated and challenged, indicating specific memory CD4^+^ T cells. Whether these cells are recognizing only HSA-derived peptides, Galα3LN-containing glycopeptides, or both remains to be determined. However, since we observed a robust isotype switching (from IgM to IgG) following immunization, characterized by significant production of IgG1, IgG2b, and, to a lesser extent, IgG3, our data strongly suggest engagement of CD4^+^ T cells via recognition of both peptides and glycopeptides presented by MHC II on the activated B cells.^84,85^ Thus we propose that production of murine anti-α-Gal IgG Abs specific to the Galα3LN glycotope in α1,3GalT-KO mice occurs through the established T cell-dependent B cell memory mechanism previously demonstrated for bacterial glycoproteins, glycopeptides, and zwitterionic polysaccharides (reviewed in ref. ^85^).

High parasitemia, uncontrolled inflammation, and necrosis of heart myocytes are some of the main factors that influence the development of myocardial dysfunction in ChD patients.^86^ In this study, histopathological analyses of heart tissue from mice vaccinated with Galα3LN-HSA+/−LMPLA showed significant downregulation of inflammation and low levels of myocyte necrosis, when compared to the HSA-LMPLA group. Furthermore, we observed a much lower infiltration of CD3^+^, CD4^+^, and CD8^+^ T cells in the cardiac tissue of animals vaccinated with Galα3LN-HSA+/−LMPLA, corroborating therefore the protective immune response generated by the two vaccine formulations.

In conclusion, here we performed the first proof-of-concept studies, which demonstrate the efficacy of two α-Gal-based vaccine formulations for the prophylactic immunization against experimental acute *T*. *cruzi* infection. To this end, we validated the α1,3GalT-KO mouse model, which closely resembles the major protective human anti-glycan response observed in ChD. This is a suitable experimental model to evaluate more effective prophylactic and therapeutic α-Gal-based vaccines.

## Methods

### Ethics statement

Human serum samples used for preparation of the ChD human serum pool (ChHSP) and for purification of anti-α-Gal Abs used in this study were obtained from chronic adult ChD patients from the Hospital Clinic of Barcelona (HCB), Barcelona Institute for Global Health (ISGlobal), Barcelona, Spain and were kindly donated by Dr. Joaquim Gascón. The Institutional Review Board (IRB) protocol was approved by the IRB Committee of HCB, and all ChD patients signed an informed consent form.

All animal procedures were performed according to NIH guidelines and the appropriate protocol (A-201211-1) approved by the Institutional Animal Care and Use Committee (IACUC) at the University of Texas at El Paso (UTEP).

### Mice

C57BL/6 α1,3-Galactosyltransferase-knockout (α1,3GalT-KO) mice^35,36^ were kindly donated by Professor Peter J. Cowan, St. Vincent’s Hospital Melbourne and University of Melbourne, Australia. Mice were bred and maintained under biosafety level 2, pathogen-free conditions at the Laboratory Animal Resources Center at UTEP. Six-to-ten-week-old female α1,3GalT-KO mice were used for all experiments. WT (α1,3GalT-WT) C57BL/6 mice (Jackson Laboratories, Bar Harbor, ME) were also used.

### Parasites and mammalian-cell culture

*T*. *cruzi* Y strain^87^ was obtained from ATCC (Manassas, VA), and the CL Brener clone,^88,89^ expressing red-shifted firefly luciferase (CL Brener-*luc*), was kindly donated by Professor John Kelly (London School of Tropical Medicine and Hygiene, UK). Mammalian TCT forms were obtained by infecting rhesus monkey kidney epithelial LLC-MK2 cells (ATCC) with either axenic metacyclic trypomastigotes or mouse-derived bloodstream trypomastigotes at a multiplicity of infection (m.o.i.) of 10 for 2 h at 37 °C. Cells were maintained in Dulbecco’s modified eagle medium (DMEM), supplemented with 10% heat-inactivated fetal bovine serum (FBS) (Gibco, Thermo Fisher Scientific, Waltham, MA) and 1% penicillin–streptomycin at 37 °C with 5% CO_2_ atmosphere. TCTs were collected, washed with sterile PBS by centrifugation (1000 × *g*, 10 min, at 4 °C), and prepared for mouse infections and other experiments. To devoid the parasites of sialic acid, infected cells were washed with DMEM with 0.2% BSA and 1% penicillin–streptomycin and incubated for 2 h, at 37 °C, as described.^22^

### Parasitemia and survival rate determination

Five μL of blood, obtained by venipuncture of the mouse tail, was diluted in 18 μL of 0.89% ammonium chloride in PBS, and TCTs (Y strain) were counted on a hemocytometer. Data were plotted as the number of TCTs per microliter of blood. Mice were evaluated for survival daily. Survival rates were plotted as Kaplan–Meier curves.

### Immunopurification of human and murine anti-α-Gal antibodies

Murine anti-α-Gal Abs were obtained from α1,3GalT-KO mice infected with CL Brener-*luc*. Immunopurification of both human and murine anti-α-Gal Abs was performed as described,^19^ using Synsorb 115 beads, containing the Galili trisaccharide Galα1,3Galβ1,4GlcNAc (Galα3LN) covalently linked to silica particles (0.88 pmol oligosaccharide/g Synsorb; ChemBiomed, Edmonton, Canada). Beads were extensively washed with PBS–0.5 M NaCl to remove non-anti-α-Gal IgG (unbound IgG). Murine anti-α-Gal Abs were eluted from the Synsorb 115 with 0.1 M glycine–HCl or 50 mM citric acid pH 2.8 and immediately neutralized with 1 M Tris, pH 8.0. Both the unbound IgG and the anti-α-Gal Ab fractions were dialyzed against PBS, overnight (O/N), at 4 °C, concentrated in a 30-kDa Amicon Ultra-15 Centrifugal Filter Unit (Millipore Sigma, Burlington, MA), and stored at −20 °C until use.

### In vitro host cell infection assays

LLC-MK2 cells were maintained as described above and Y strain TCTs were used for in vitro infection. Briefly, cells were seeded on 15-mm microscope coverslips in 24-well tissue culture plates (Corning Costar, Thermo Fisher Scientific). After incubation at 37 °C in humidified atmosphere containing 5% CO_2_, O/N, non-adherent cells were washed off. Adherent cells were infected with Y strain TCTs (m.o.i. = 10) for 2 h, at 37 °C. During infection, purified murine anti-α-Gal IgG Abs were added at 100, 10, and 1 μg/mL. Unbound IgG fraction (containing non-anti-α-Gal IgG Abs) and medium alone were used as negative controls. After infection, parasites in the cell culture medium supernatant were counted for 8 consecutive days. Infected adherent cells were then fixed in 4% formaldehyde and permeabilized with 0.2% Triton X-100 in PBS for 5 min on ice. Cells were washed and blocked with 1% BSA–PBS for 30 min at room temperature (RT). Internalized parasites were stained with 1 μg/mL of 4,6-diamidino-2-phenylindole (Invitrogen, Carlsbad, CA) and immunolabeled with purified murine anti-α-Gal Abs (1 μg/mL) for 2 h at RT. Reactivity of murine anti-α-Gal IgG with intracellular parasites was detected by incubation with goat anti-human IgG (2.2 μg/mL) (Thermo Fisher Scientific) for 1 h, at RT, followed by Alexa Fluor 488 donkey anti-goat IgG (10 μg/mL) (Thermo Fisher Scientific) for 1 h at RT. Visualization of at least 1000 cells was done using a BD Pathway 855 Imager (BD Bioscience). Experiment was performed in triplicate determinations.

### Preparation of liposomes and Galα3LN-HSA immunizations

Liposomal-monophosphoryl lipid A (LMPLA) were prepared as described.^65^ Briefly, 1,2-dimyristoyl-sn-glycero-3-phosphocholine, cholesterol, and 1,2-dimyristoyl-sn-glycero-3-phospho-rac-(1-glycerol) (Avanti Polar Lipids, Alabaster, AL) were dissolved in freshly distilled chloroform stabilized with 0.75% ethanol at a molar ratio of (9:7.5:1) along with monophosphoryl lipid A (Sigma-Aldrich, St. Louis, MO) for a final concentration of 100 μg/mL. Liposomes were dried under nitrogen stream and placed O/N in a desiccator containing silica-gel desiccant beads (Thermo Fisher Scientific). Liposomes were then resuspended for a final concentration of 10 μg/200 μL of sterile PBS, pH 7.4, containing 20 μg Galα3LN-HSA (3-atom spacer, NGP2334, Dextra Laboratories, Reading, UK) or 20 μg recombinant HSA (Thermo Fisher Scientific), then filtered using a 0.2-μm disc filter (Thermo Fisher Scientific). For immunizations in the absence of LMPLA, a stock solution of 1 mg/mL Galα3LN-HSA in sterile, deionized milli-Q water was prepared and kept in 200-μL aliquots at −20 °C until use.

### Immunizations and parasite challenge

Groups of 6-to-10-week-old female C57BL/6 α1,3GalT-KO mice (*n* = 6 per group) were immunized i.p. with 20 μg/dose/mouse of Galα3LN-HSA alone (Galα3LN-HSA), in combination with 10 μg/dose/mouse of LMPLA adjuvant (Galα3LN-HSA+LMPLA), or with the carrier protein and adjuvant alone (HSA+LMPLA) as the control group. A naive group was also included and handled like the experimental groups. Three days before prime (N), blood was collected from all groups to obtain baseline serum levels. Immunizations were achieved by one prime (P) and three boost immunizations (B1, B2, and B3), i.p., at 7-day intervals, where LMPLA adjuvant was only administered during P and B1 immunizations for the Galα3LN-HSA+LMPLA and HSA+LMPLA groups. All groups were challenged i.p. with 1 × 10^5^ CL Brener-*luc* TCTs 3 weeks after B3. Animals were humanely euthanized at the experimental endpoint (EP), 32 days after parasite challenge. Serum was collected between immunizations and throughout the experiment for further analyses. Weight was closely monitored and mice were examined for signs of toxicity and abnormal behavior. Survival of experiment was recorded up to experimental endpoint.

### In vivo whole-body parasite bioluminescence imaging

Mice were analyzed for whole-body parasite burden using IVIS Lumina III In Vivo Imaging System (PerkinElmer, Waltham, MA), as described.^40^ Previous to imaging, mice were injected i.p. with 150 mg/kg D-luciferin, potassium salt (Gold Biotechnology, St. Louis, MO), in 100 μL PBS, and anesthetized soon after with 2% isofluorene gas with oxygen. Live animal images were acquired in the automatic exposure setting 10 min after D-luciferin injection.

### Anti-α-Gal Ab titers and Ab isotype determination by CL-ELISA

To determine the levels of anti-α-Gal Abs in naive and TCT-infected α1,3GalT-KO and α1,3GalT-WT mice, 96-well CL-ELISA microplates (Thermo-Fisher Scientific) were immobilized with 5 µg/mL (500 ng/well) of Galα3LN-BSA (3-atom spacer, NGP0334, Dextra Laboratories, Reading, UK) in 0.2 M carbonate–bicarbonate buffer, pH 9.6 (CB buffer). Wells were blocked with 1% BSA in PBS, pH 7.4 (1% BSA-PBS), for 1 h at 37 °C. Mouse sera (at 1:100 dilution) in 1% BSA–PBS was added and incubated at 37 °C for 1 h. Microplates were then sequentially incubated with 50 µL of each goat anti-mouse IgG (1 µg/mL; eBioscience, Thermo Fisher Scientific), donkey anti-goat IgG-biotin conjugate (1 µg/mL; eBioscience, Thermo Fisher Scientific), and streptavidin–horseradish peroxidase (HRP) (1:2000; Zymed) for 1 h at 37 °C. Microplates were washed 4× with 200 µL PBS–0.05% Tween 20 (PBS-T) between incubation steps. For detection of Ab–antigen complexes, the luminescent reagent (50 µL, at 1:20 dilution in CB buffer; ECL, Pierce, Thermo Fisher Scientific) was added and relative luminescence units were measured using a Luminoskan Ascent luminometer (eBiosystems, Thermo Fisher Scientific).

Anti-α-Gal Ab titers in the sera of mice immunized with Galα3LN-HSA+/−LMPLA were determined by CL-ELISA,^29^ as detailed in Methods. First, antigen and serum pools were cross-titrated to optimize the immunoassay. Galα3LN-HSA was immobilized at 125 ng/well in CB buffer. Free binding sites were blocked with 5% skim milk in PBS), for 1 h at 37 °C. Mouse sera collected at various timepoints throughout study (Fig. 2) were used as primary Abs at 1:100 dilution in PBS-T), for 1 h at 37 °C. Donkey anti-mouse IgG biotin-conjugated Ab (1:2000 dilution) was used as secondary Ab, followed by High Sensitivity NeutrAvidin-HRP enzyme (1:5000 dilution) (both conjugates were from Thermo Fisher Scientific), both for 1 h at 37 °C. CL-ELISA was developed with SuperSignal ELISA Pico Chemiluminescent Substrate, at 1:8 dilution in CB buffer with 0.1% BSA to reduce the nonspecific luminescence,^90^ using a Luminoskan luminometer (both from Thermo Fisher Scientific). Microplates were washed 3× with 200 µL PBS-T between incubation steps except before blocking.

Ab isotype levels of IgG1, IgG2b, IgG3, IgM, IgA, and IgE specific to Galα3LN-HSA were also analyzed by CL-ELISA using the same protocol and specific isotypes as secondary Abs conjugated to HRP (goat anti-mouse IgG1-HRP, goat anti-mouse IgG2b-HRP, and goat anti-mouse IgG3-HRP, goat anti-mouse IgM-HRP, goat anti-mouse IgA-HRP, and rat anti-mouse IgE-HRP; Abcam, Cambridge, MA).

For IgM, IgA, and IgE measurement, prior to the CL-ELISA we depleted IgG Abs by preincubating each serum sample with a 15-μL bead suspension mixture of protein A-Sepharose 4B and protein G-Sepharose (A:G, 1:1, v/v), both fast flow (Millipore-Sigma, St. Louis, MO), and recombinant HSA (1 mg/mL), for 1 h at 37 °C in rotatory shaker.

### Quantitative real-time PCR (qPCR) analysis of parasitic load in tissues

At experimental endpoint, mice were euthanized by CO_2_ overdose, and several organs were harvested for semiquantitative parasite load quantification.^91^ Briefly, genomic DNA was extracted from 20–30 mg of tissue using the High Pure PCR Template Preparation Kit (Roche Diagnostics, Indianapolis, IN), following the manufacturer’s instructions. Before extraction, tissue was homogenized in sterile, ice-cold PBS using sterile gentleMACS M tubes on the gentleMACS dissociator (Miltenyi Biotec, Auburn, CA), running in the protein program mode. All samples were spiked with 5 μL of a 40 pg/μL of linearized pUC57 plasmid, containing the following sequence (base pairs 434–614) from *Arabidopsis thaliana* tonoplast intrinsic protein 5;1 (TIP5;1) gene (accession number: NM_114612), as an internal amplification control (IAC)^92^: 5′–ACCGTCATGGAACAGCACGTACCGATTTATAAGATTGCTGGAGAAATGACTGGATTTGGAGCATCTGTTCTTGAAGGTGTTTTAGCTTTCGTCTTGGTTTATACTGTGTTCACGGCTAGCGATCCCAGACGTGGGCTACCTTTAGCAGTGGGACCTATATTTATAGGGTTTGTTGCGGGAG–3′. After DNA extraction, all samples were diluted to 20 ng/μL and a total of 100 ng of DNA was used for a final volume of 20 μL. Amplification of *T*. *cruzi* satellite DNA was done by using the specific primers^93,94^ Cruzi 1 (5′–ASTCGGCTGATCGTTTTCGA–3′) and Cruzi 2 (5′–AATTCCTCCAAGCAGCGGATA–3′), both at 750 nM, and the TaqMan probe Cruzi 3 (6FAM–CACACACTGGACACCAA–MGB–NFQ) at 50 nM. Both the forward (5′–ACCGTCATGGAACAGCACGTA–3′) and the reverse (5′–CTCCCGCAACAAACCCTATAAAT–3′) primers for the IAC were used at 100 nM, and the TaqMan probe (VIC–AGCATCTGTTCTTGAAGGT–MGB–NFQ) was used at 50 nM. Standard curves were done with a spiked pool of naive tissue lysate (heart, spleen, colon, stomach, lungs, liver, intestine, and skeletal muscle) with 1 × 10^5^ parasites/mL and diluting 1/10 in naive mouse DNA. PCR conditions were as detailed above and consisted of 50 °C for 2 min, 94 °C for 10 min, followed by 40 cycles at 95 °C and 58 °C for 1 min; fluorescence was collected after each cycle. All samples were run in duplicate in Step One Plus Real Time PCR System (Applied Biosystems, Foster City, CA).

### α-Galactosidase treatment

First, MaxiSorp Nunc polystyrene microplate (Thermo Fisher Scientific) wells were coated with 125 ng/well of Galα3LN-HSA in CB buffer and incubated O/N at 4 °C. The microplate free sites were blocked with 200 µL 5% skim milk–PBS for 1 h at 37 °C, washed three times with 150 µL PBS-T, and subsequently washed twice with 150 µL of 100 mM potassium phosphate buffer (pH 6.5), to equilibrate the microplate to the appropriate pH before enzyme addition. Then green coffee bean α-galactosidase (in ammonium sulfate suspension, ≥9 units/mg protein; G8507, Sigma-Aldrich) was centrifuged at 10,000 × *g* for 10 min at 4 °C to remove excess ammonium sulfate. The supernatant was discarded and the pellet containing the enzyme was gently resuspended in ice-cold 100 mM potassium phosphate buffer (pH 6.5). Fifty microliters of the enzyme solution (0.5 U/well) were added to each well, and the plate was incubated for 24 h at 37 °C in a humid chamber. The microplate was washed twice with 200 µL PBS-T and CL-ELISA was performed as described above. The reduction of reactivity of specific IgG Abs elicited in mice immunized with Galα3LN-HSA or Galα3LN-HSA+LMPLA was compared against enzyme-untreated wells that were incubated with the 100 mM potassium phosphate buffer under the same conditions.

### Parasite lysis assay

Y strain or CL Brener-*luc T*. *cruzi* TCTs (20 μL of 1 × 10^6^–10^7^ parasites/mL in DMEM, pH 7.4) were preincubated with 1, 10, or 100 μg of purified murine anti-α-Gal Abs, obtained as described above. In parallel, 2 or 20 μL of serum from mice immunized with Galα3LN-HSA or Galα3LN-HSA+LMPLA was also incubated in the presence of 100 mM MgCl_2_ (to reduce parasite sialic acid inhibitory effect on anti-α-Gal Abs^22^), for 30–60 min, at 37 °C, under a humidified atmosphere of 5% CO_2_. As controls, TCTs in PBS (negative control, live), TCTs preincubated with 4% formaldehyde (positive control, dead), and NMS (negative control) at the same incubation conditions. Purified human anti-α-Gal Abs from chronic ChD patients (Ch anti-α-Gal Abs, at 20 μg/mL), obtained as described^19^ and detailed above, was incubated with TCTs in the presence of 100 mM MgCl_2_, under the same conditions. After incubation, samples were loaded onto a hemocytometer (Thermo Fisher Scientific) and both live and dead parasites were counted in triplicate determinations.

### Immunoaffinity purification of murine anti-α-Gal antibodies obtained by immunization with Galα3LN-HSA+/−LMPLA

Galα3LN-HSA (50 μg) was spotted and air-dried onto nitrocellulose membrane strips (Thermo Fisher Scientific) and incubated for 1 h at 37 °C in PBS, under gentle agitation using a rotatory shaker. Membrane was then blocked with 1% BSA–PBS for 1 h at 37 °C on shaker and washed three times with PBS. Then serum pools from mice immunized with Galα3LN-HSA, Galα3LN-HSA+LMPLA, or HSA+LMPLA (control group), or from naive animals, at day −7, were diluted 1:10 in PBS and added to the blocked nitrocellulose membrane. The membrane was incubated O/N at 4 °C on rotatory shaker, then washed three times with PBS. Bound Abs were eluted with 50 mM citric acid, pH 2.8, and immediately neutralized with 1 M Tris-HCl, pH 8.0. Eluates were dialyzed by centrifugation in a 30-kDa Amicon Ultra-15 Centrifugal Filter Unit (Millipore Sigma) and washed with PBS. Ab concentration was measured with the Pierce BCA Protein Assay Kit (Thermo Fisher Scientific) and stored at 4 °C in PBS–0.1% sodium azide until use. We obtained 306, 534, 741, and 302 μg/mL of Abs from the Galα3LN-HSA, Galα3LN-HSA+LMPLA, HSA+LMPLA, and naive groups at day −7, respectively.

### Confocal immunofluorescence microscopy

CL Brener-*luc* TCTs from 4–6-day-infected LLC-MK2 cells were collected in DMEM–0.2% BSA (no FBS) for 2 h at 37 °C, at 5% CO_2_, to devoid parasites of sialic acid added via TS action.^22^ After incubation, 1 × 10^6^ parasites were air-dried on each well of a Nunc 4-well chambered slide (Thermo Fisher) and immediately fixed with 500 μL of ice-cold methanol for 5 min, at −20 °C. Chambered slides were gently washed three times with 500 μL PBS. Parasites were then blocked O/N at 4 °C with 500 μL of 2% BSA in PBS. Parasites were washed three times with 500 μL PBS, then purified Ch anti-α-Gal and murine Ch anti-α-Gal IgG Abs were added at 1 μg/mL in 500 μL 0.1% BSA, and incubated O/N at 4 °C. Parasites were washed three times with 500 μL PBS, then incubated with goat anti-mouse IgG (H+L) Highly Cross-Adsorbed Secondary Ab Alexa Fluor™ 594 and isolectin GS-IB_4_ from *Griffonia simplicifolia* labeled with Alexa Fluor™ 488 (both from Thermo Fisher Scientific), at 5 μg/mL, for 1 h at RT, protected from light. Finally, parasites were washed three times with 500 μL PBS and chambers were removed from slides, allowed to air-dry, mounted with a drop of Vectashield antifade mounting medium (Vector Laboratories, Burlingame, CA), and coverslips were sealed with nail polish. Images were captured using a Zeiss LSM 700 confocal microscope equipped with ×63 oil immersion objective; images were acquired using 1-Airy unit and 1024-by-1024-pixel resolution. Zen 2009 software was used to capture and analyze images (Zeiss, Oberkochen, Germany).

### Analysis of serum cytokines

For comparing the serum cytokine profile of α1,3GalT-KO versus α1,3GalT-WT mice infected with TCTs, serum was collected from both groups in a period of 4 weeks of infection. Cytokine measurements (IFN-γ, TNF-α, IL-2, IL-4, and IL-10) were performed by sandwich ELISA and compared to commercial standard curve according to the manufacturer (BD Biosciences, Franklin Lakes, NJ). Vaccinated mouse serum cytokines and chemokines (Eotaxin (or CCL11), G-CSF, GM-CSF, IFN-γ, IL-1α, IL-1β, IL-2, IL-3, IL-4, IL-5, IL-6, IL-7, IL-9, IL-10, IL-12 (p40), IL-12 (p70), IL-13, IL-15, IL-17, IP-10 (or CXCL10), KC-like (or CXCL1), LIF, LIX (or CXCL5), MCP-1 (or CCL2), M-CSF, MIG (or CXCL9), MIP-1α (or CCL3), MIP-1β (or CCL4), MIP-2 (or CXCL2), RANTES (or CCL5), TNF-α, and VEGF) were analyzed using the MILLIPLEX MAP Mouse Cytokine/Chemokine Magnetic Bead Panel Premixed 32 Plex using the manufacturer’s protocol (EMD Millipore, Billerica, MA). Briefly, sera obtained from experimental endpoint (32 dpi) were diluted 1:2 and subjected to analysis by fold change against the challenged-only group. Results are representative of two biological replicates, and all determinations were performed in duplicate.

### CD4^+^ and CD8^+^ T cell analysis by flow cytometry

Splenocytes were harvested from α1,3GalT-KO mice after immunizations and at experimental endpoint (Fig. 2a) in 10 mL of ACK red blood cell lysis buffer (0.83% ammonium chloride, 0.1% potassium bicarbonate, 0.04% EDTA, pH 7.4) and washed in freshly prepared complete DMEM (DMEM+10% heat-inactivated FBS+1% penicillin–streptomycin) with addition of 50 μM 2-mercaptoethanol. Cells were seeded in 12-well flat-bottom plates (Corning, Thermo Fisher Scientific) and stimulated with 20 μg/mL Galα3LN-HSA and incubated for 24 h at 37 °C, with 5% CO_2_. After incubation, supernatant was removed and cells were washed with sterile PBS containing 1% BSA and 0.09% sodium azide (PBSA/Az). The FcγR was blocked with 10% fresh mouse serum in PBS and incubated on ice for 15 min. After a PBSA/Az wash, cells were resuspended in complete DMEM and cells were stained with fluorochrome-conjugated Abs PE-Cy7-labeled anti-CD3e, PE-labeled anti-CD4, FITC-labeled anti-CD8, and APC-labeled anti-CD44 (all conjugates from BD Bioscience, San Jose, CA and Tonbo Biosciences, San Diego, CA) for 30 min at 4 °C, protected from light. After another wash with PBS/Az, cells were fixed with 1% paraformaldehyde and transferred to FACS tubes. A total of 50,000 events were analyzed by flow cytometry using the Gallios Flow Cytometer and analyzed with the Kaluza Analysis Software (Beckman Coulter, Brea, CA). Gates were set for lymphocyte cells (CD3e-PE-Cy7 labeling) using forward and side scatter properties, and the percentages of activated CD4^+^ and CD8^+^ T cells were obtained on gated CD3^+^ T cells (Supplementary Figure 3).

### Histopathology

Hearts from animals immunized with Galα3LN-HSA or Galα3LN-HSA+LMPLA and from the control groups (HSA+LMPLA and naive) were harvested and immediately cut longitudinally and fixed in 4% paraformaldehyde (Thermo Fisher Scientific), O/N at 4 °C. Then tissue sections were sequentially incubated in 15% and 30% sucrose in PBS, for 48 h at 4 °C. Samples were then embedded in paraffin, sectioned, stained with hematoxylin and eosin, and analyzed at MD Anderson Cancer Center (MDACC) Science Park Research Histology, Pathology & Imaging Core (Smithville, TX). In each section of the heart, the infiltration of mononuclear cells in the left ventricle from 25 fields (at ×400) was manually counted and analyzed. Moreover, heart sections were stained for CD3^+^ T cells with rat anti-human CD3 mAb (clone CD3–12, Serotec/Bio-Rad), which cross-reacts with mouse CD3; for CD4^+^ T cell with rat anti-mouse CD4 mAb (clone 4SM95, eBioscience, Invitrogen); and for CD8^+^ T cell with rat anti-mouse CD8 mAb (clone 4SM15, eBioscience, Invitrogen). Images were acquired using an Aperio ScanScope CS Digital Pathology Scanner (Leica Biosystems, Buffalo Grove, IL).

### Statistical analysis

Data points are presented as the average of triplicate determinations with their corresponding standard error of the means (S.E.M.). Kaplan–Meier survival rate curves, Student’s *t* test, one-way analysis of variance (ANOVA), or two-way ANOVA were employed in the statistical analysis, as indicated in the figure legends. Graphs and statistical analysis were achieved using the Graph Pad Prism 7 Software (GraphPad Software, Inc., La Jolla, CA).

### Reporting summary

Further information on research design is available in the Nature Research Reporting Summary linked to this article.

## Supplementary information

Supplementary Information.

Life Sci Reporting Summary

## Data Availability

All data that support the findings of this study are available from the corresponding author upon reasonable request.

## Supplementary information

**Supplementary information** accompanies the paper on the *npj Vaccines* website (10.1038/s41541-019-0107-7).
