# Supplementary material for: A prophylactic α-Gal-based glycovaccine effectively protects against murine acute Chagas disease
Source: NPJ Vaccines. 2019 Mar 22;4:13. doi: 10.1038/s41541-019-0107-7 (PMC6430786; doi:10.1038/s41541-019-0107-7)
Supplement: Supplementary file 1 — Supplementary Information. [file 41541_2019_107_MOESM1_ESM.pdf]

Supplementary Information

Supplementary Table 1. Expression of cytokines, chemokines, and growth factors in the serum of α1,3GalT-KO mice immunized with Galα3LN-HSA, Galα3LN-HSA+LMPLA, or HSA+LMPLA, and challenged with *T. cruzi* TCTs.

Immunization group	Immunized-challenged, mean (picograms/mL)	SD	Challenged only, mean (picograms/mL)	SD	*p* value [a]	Fold-change [b]
**Galα3LN-HSA**						
IL-1α	143.50	19.40	465.70	5.60	0.000026	0.3
IL-4	0.82	0.20	1.95	0.10	0.002772	0.4
IL-6	39.92	4.10	71.75	1.60	0.000525	0.6
IL-12 p70	29.76	4.00	50.07	0.90	0.002555	0.6
LIF	4.49	0.20	6.38	0.30	0.000754	0.7
MCP-1 (CCL2)	114.20	19.86	234.60	3.50	0.001295	0.5
MIP-1α (CCL3)	53.06	3.09	66.50	1.70	0.005246	0.8
RANTES (CCL5)	58.91	5.01	85.76	2.60	0.002377	0.7
KC (CXCL1)	111.10	26.01	207.70	9.25	0.008348	0.5
LIX (CXCL5)	593.50	114.97	1011.00	126.04	0.014973	0.6
MIG (CXCL9)	951.10	96.96	1641.00	19.80	0.000707	0.6
G-CSF	86.23	5.29	232.40	0.00	0.000003	0.4
VEGF	2.94	0.14	3.32	0.09	0.029723	0.9
**Galα3LN-HSA+LMPLA**						
IL-1α	110.60	7.20	465.70	5.60	<0.000001	0.2
IL-2	57.85	10.50	18.37	0.20	0.007487	3.1
IL-4	4.11	0.70	1.95	0.10	0.015703	2.1
IL-9	491.80	46.50	243.40	24.10	0.002419	2.0
IL-12 p40	20.31	3.90	30.81	0.00	0.022701	0.7
IL-15	187.40	12.50	143.10	4.80	0.009889	1.3
MCP-1 (CCL2)	137.30	40.37	234.60	3.50	0.032521	0.6
MIP-1α (CCL3)	97.12	6.18	66.50	1.70	0.002846	1.5
MIP-1β (CCL4)	67.49	13.03	100.70	1.84	0.027568	0.7
RANTES (CCL5)	56.05	9.49	85.76	2.60	0.014567	0.7
MIG (CXCL9)	994.30	194.90	1641.00	19.80	0.011540	0.6
G-CSF	95.12	12.13	232.40	0.00	0.000112	0.4
VEGF	13.40	4.82	3.32	0.09	0.049323	4.0
**HSA+LMPLA**						
IL-1α	86.50	10.10	465.70	5.60	0.000001	0.2
IL-6	41.81	4.20	71.75	1.60	0.000730	0.6
IL-12 p70	33.44	5.40	50.07	0.90	0.014692	0.7
IL-12 p40	18.32	4.70	30.81	0.00	0.024089	0.6
IL-17	3.84	0.40	4.83	0.13	0.037465	0.8
LIF	9.12	0.90	6.38	0.30	0.015019	1.4
MCP-1 (CCL2)	130.20	33.11	234.60	3.50	0.013730	0.6
MIP-1β (CCL4)	70.94	13.74	100.70	1.84	0.044984	0.7
MIG (CXCL9)	1230.00	180.91	1641.00	19.80	0.038901	0.7

[a] Student's *t*-test was used for comparing animals immunized with Galα3LN-HSA+/-LMPLA or HSA+LMPLA, followed by parasite challenge, with a group of animals subjected to challenge only. Cytokines, chemokines, and growth factors were measured at the experimental endpoint (32 dpi) in two biological replicates, each containing two technical replicates, as described in Methods section.

[b] Ratio of protein levels of immunized-challenged group to challenged-only group. The red-to-green gradient indicates fold-change from lower to higher values. Only statistically significant reduction or increase in protein levels in the immunized-challenged group in comparison with challenged-only group are shown.

Supplementary Figures

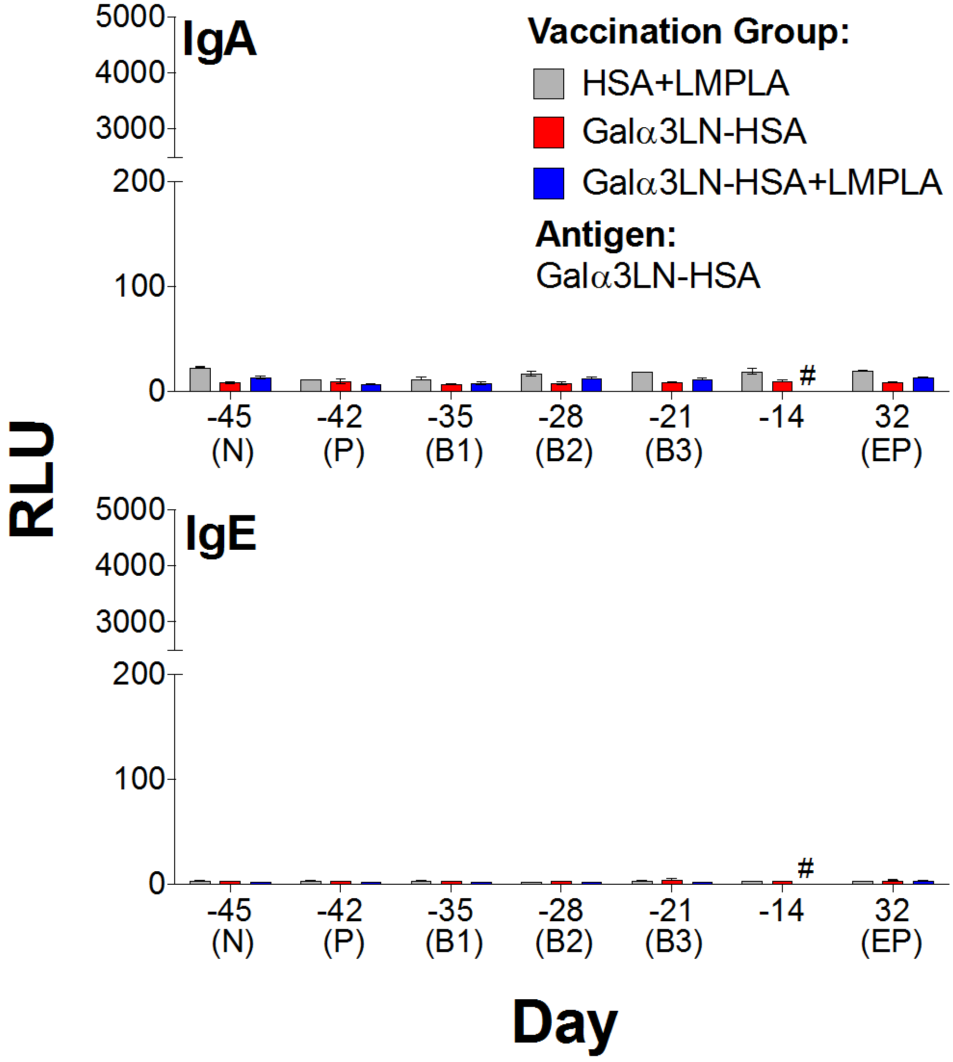

Supplementary Figure 1. Kinetics of IgE and IgA elicited by immunization with Galα3LN-HSA, Galα3LN-HSA+LMPLA, or HSA+LMPLA. Prior to the CL-ELISA, each serum sample was pre-incubated with a 15-μL bead suspension mixture of protein A-Sepharose 4B and protein G-Sepharose, both fast flow (A:G, 1:1, v/v), and recombinant HSA (1 mg/mL), for 1 h at 37°C. Reactivity of mouse sera from α1,3GalT-KO mice immunized with Galα3LN-HSA, Galα3LN-HSA+LMPLA, or HSA+LMPLA, was evaluated by CL-ELISA, using Galα3LN-HSA as immobilized antigen. Error bars indicate S.E.M. of triplicate determinations. Samples no longer available are indicated (#).

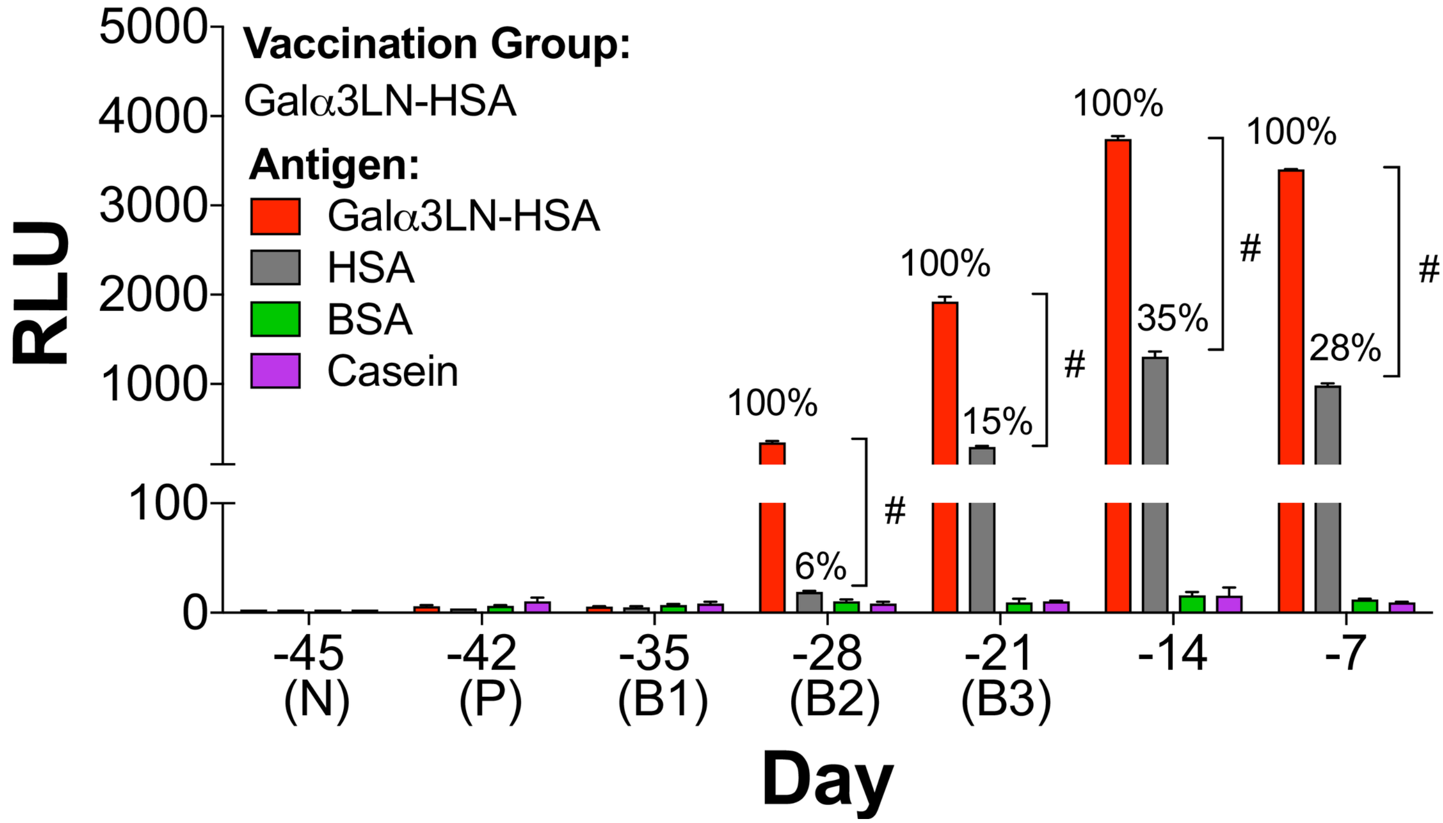

Supplementary Figure 2. Kinetics of background response to different antigens (Galα3LN-HSA, HSA, BSA, and casein) elicited by immunization with Galα3LN-HSA. CL-ELISA was performed as described in Methods. Error bars indicate S.E.M. of triplicate determinations. The α-Gal-specific reactivity is indicated (#).

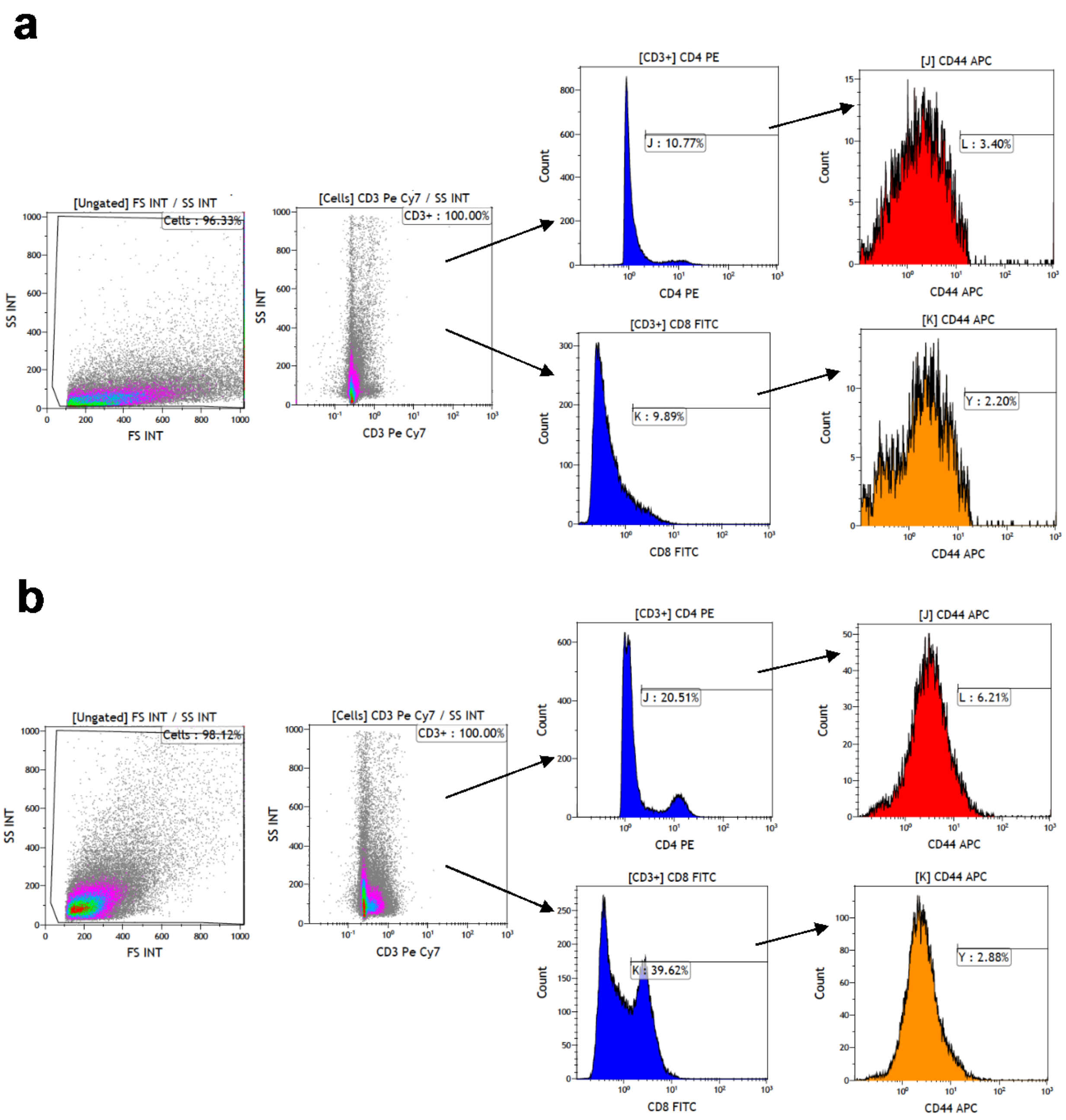

Supplementary Figure 3. Gating strategies used for flow cytometry. (**a**) Gating strategy was performed with samples from a group of unstimulated naïve mice (α1,3GalT-KO C57Bl/6) using forward and side scatter properties to eliminate debris. $CD4^+$ and $CD8^+$ T cells were both pre-gated with $CD3^+$, and $CD44^+$ T cells were pre-gated with $CD3^+CD4^+$ or $CD3^+CD8^+$ T cells. The same gating strategy set for naïve samples was applied to all experimental samples shown in manuscript. (**b**) Example of experimental sample of a group of mice (α1,3GalT-KO C57Bl/6) immunized with Galα3LN+HSA and infected with *T. cruzi*, then splenocytes were stimulated ex-vivo with Galα3LN+HSA antigen as described in Methods section

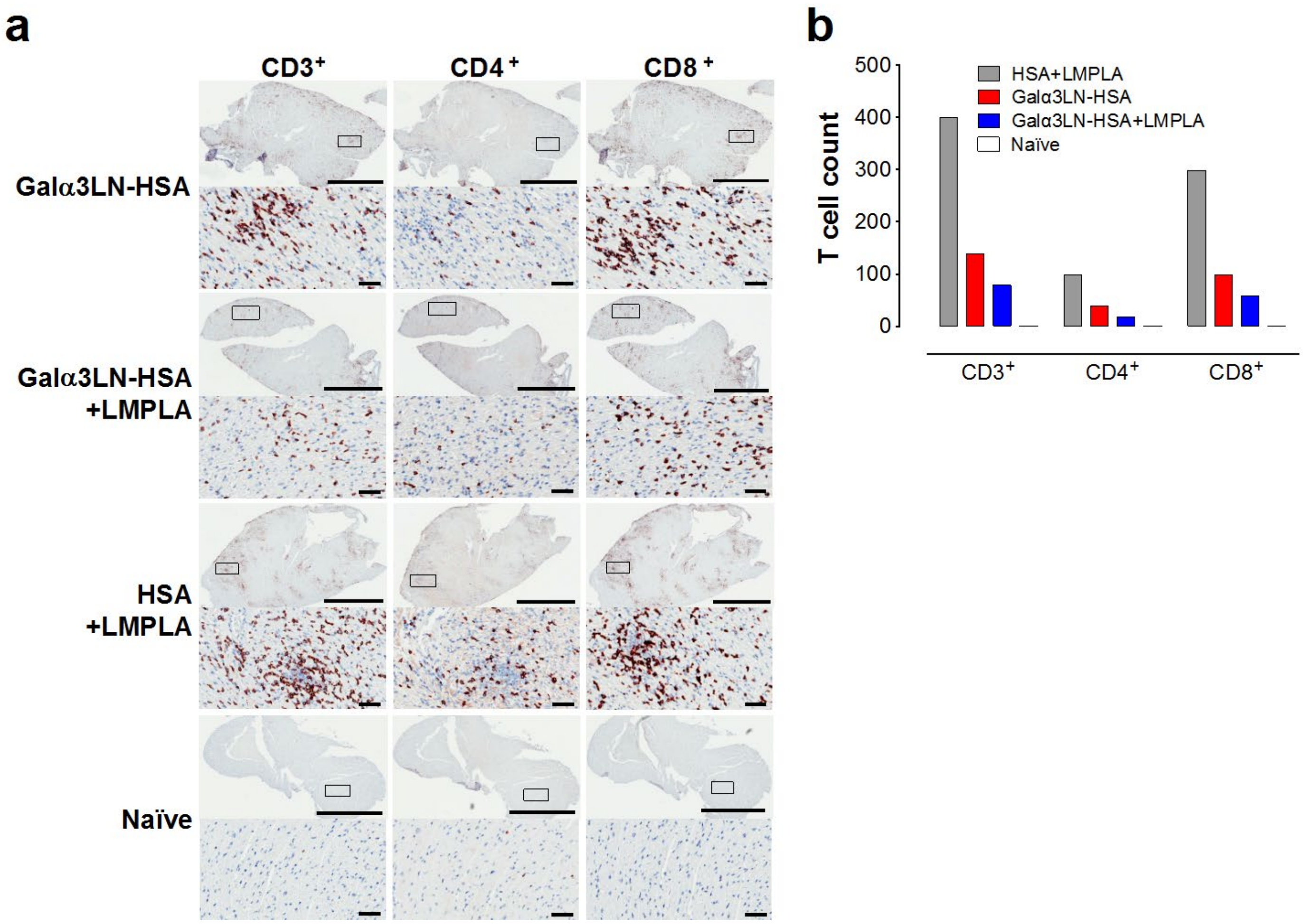

Supplementary Figure 4. Representative immunohistochemistry analysis of $CD3^+$, $CD4^+$, and $CD8^+$ T cells in the cardiac tissues at experimental endpoint. (**a**) Specific antibody staining of $CD3^+$, $CD4^+$, and $CD8^+$ T cells. In each animal group, the top panels show the whole tissue sections, at 1.5x magnification (long horizontal bar: 2 mm); the bottom panels indicate zoomed sections from rectangular insets in the top panels, at 40x magnification (short horizontal bar: 50 μm). (**b**) Quantitative analysis of one representative mouse from each group. For consistency, only ventricle areas were evaluated. Predominant inflammatory cells were $CD3^+$ T cells, and $CD8^+$ T cells were higher than $CD4^+$ T cells present in the sections examined.
